# Exploring the Environmental Sustainability of Primary Al–Air Batteries for Long‐Term Energy Storage Applications

**DOI:** 10.1002/cssc.202502714

**Published:** 2026-04-19

**Authors:** Hüseyin Ersoy, Manuel J. Baumann, Friedrich B. Jasper, Christina Wulf, Marcel Weil, Tomás B. Ramos, Stefano Passerini

**Affiliations:** ^1^ Institute for Technology Assessment and Systems Analysis (ITAS) Karlsruhe Institute of Technology (KIT) Karlsruhe Germany; ^2^ Center for Environmental and Sustainability Research (CENSE), NOVA School of Science and Technology NOVA University Lisbon (UNL) Caparica Portugal; ^3^ Institute of Climate and Energy Systems (ICE) Forschungszentrum Jülich (FZJ) Jülich Germany; ^4^ Helmholtz Institute Ulm for Electrochemical Energy Storage (HIU) Karlsruhe Institute of Technology (KIT) Ulm Germany; ^5^ Center for Transport Technologies Austrian Institute of Technology GmbH (AIT) Vienna Austria

**Keywords:** aluminum–air battery, LCA, long‐term energy storage, metal fuels, power‐to‐metal

## Abstract

The transition toward a decarbonized energy system requires long‐term energy storage (LTES) solutions capable of complementing hydrogen‐based technologies. This study presents an exploratory life cycle assessment (LCA) of a primary aluminum–air battery (AAB) system as a prospective solid‐state LTES option, benchmarked against gaseous hydrogen (GH_2_) with underground storage and liquid hydrogen (LH_2_) with cryogenic tank. The AAB is evaluated under current and prospective aluminum production scenarios across different geographic contexts, and is benchmarked against alternatives using identical supply chain and use‐phase assumptions. AAB system achieves round‐trip efficiencies of 29–35%, exceeding GH_2_ and LH_2_ by at least 2% and 10%, respectively. Consequently, GH_2_ outperforms AAB across all categories on a cradle‐to‐use basis only thanks to underground storage, while AAB showing competitive performance it performs better than LH_2_ in global warming potential (GWP_100_) impact category. The conducted uncertainty analysis reveals that AAB might outperform H_2_ in GWP and eutrophication potential (freshwater) under favorable conditions. Overall, the findings highlight trade‐offs realizing climate benefits while mitigating resource and ecosystem impacts. Advancing low‐carbon smelting, material circularity, optimized logistics, and durable low‐impact components will be essential for enabling AAB to serve as a sustainable complement or partial substitute for hydrogen‐based LTES in future low‐carbon energy systems.

## Graphical abstract

An exploratory life cycle assessment compares aluminum–air batteries with gaseous and liquefied hydrogen for long‐term energy storage. The results reveal strong trade‐offs between climate benefits, resource burdens, and system efficiency, highlighting key hot spots and showing how decarbonized smelting and circular material flows can improve the competitiveness of aluminum‐based storage.

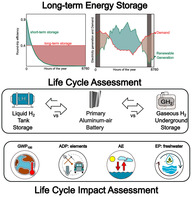

## 1 Introduction

The global energy transition represents a pivotal pathway to mitigating climate change and establishing a decarbonized energy system grounded in renewable sources [1]. Achieving this transition requires not only extensive renewable generation capacities but also the development of sustainable and flexible energy storage technologies capable of balancing supply and demand across timescales from seconds to months [2]. While batteries currently dominate short‐ and medium‐term storage applications due to their maturity and high efficiency, they remain economically and materially constrained for long‐duration applications [3]. Hence, the development of other long‐term energy storage (LTES) technologies has become an indispensable element of the future energy landscape [4]. Among various LTES candidates, hydrogen (H_2_) and synthetic fuels are widely considered for large‐scale LTES applications. Particularly, use of H_2_ in LTES remains constrained by several factors, including low volumetric energy density and energy‐intensive production and handling (e.g., compression and liquefaction) processes. Furthermore, challenges in transportation and storage hinder its wide adoption [5, 6, 7]. The limitations for the widespread deployment of energy carriers such as H_2_ have stimulated research into alternatives capable of storing renewable electricity in dense, transportable, and stable forms. In this context, metal‐based energy carriers, particularly aluminum (Al), are gaining attention. Aluminum offers several advantages, including global abundance, high volumetric energy density (23.5 kWh L^−1^), long‐term stability, and well‐established global supply chains which eliminate the need for costly infrastructure [8, 9, 10]. Furthermore, aluminum can be produced electrochemically, allowing it to function as a solid‐state energy carrier, effectively storing renewable power [11]. The produced aluminum can be utilized through direct air combustion or steam oxidation after activation, or alternatively employed as an anode material in electrochemical energy conversion systems such as aluminum–air batteries (AABs), where it can generate heat, electricity, and hydrogen, depending on the selected conversion pathway [12]. The resulting oxides upon discharge can be used for regeneration of aluminum, illustrating a circular material flow. The aluminum‐steam oxidation pathway provides round‐trip efficiencies (RTE) of about 36% [4]. While several studies have demonstrated that aluminum can provide energy storage services with competitive techno‐economic performance, research on its environmental sustainability remains limited in scope. Existing assessments primarily focus on aluminum‐water splitting for hydrogen production, emphasizing particularly the high potential of secondary aluminum as a sustainable feedstock for this application due to low energy intensity (ca. 5% of primary aluminum production energy requirement) [13, 14, 15, 16]. On the environmental dimension of sustainability, a major challenge identified is the high carbon footprint for aluminum's use as an energy carrier as discussed in Section 3.1. The high environmental impacts of primary aluminum, particularly the Hall–Héroult (HH) process due to direct greenhouse gas (GHG) emissions, motivates the ongoing advances in ‘inert anode’ technologies, and low‐carbon smelting processes are expected to substantially reduce the carbon footprint of aluminum production, thus enhancing its viability as a sustainable energy vector [17, 18]. The term ‘inert anode’ refers to electrochemically stable electrodes that ideally do not undergo dissolution or participate in parasitic side reactions during operation [18]. As recently demonstrated by ELYSIS joint venture, the inert anodes are eliminating the direct GHG emissions (CO_2_ as well as perfluorocarbon [PFC] emissions) associated with the use of conventional carbon anodes effectively at a commercial scale [19].

Beyond combustion‐based concepts such as aluminum‐steam oxidation, the direct electrochemical conversion of aluminum through AABs has emerged as a particularly promising technology [20, 21, 22, 23]. However, rechargeable AABs remain technologically immature, primarily due to issues of self‐corrosion, parasitic hydrogen evolution, and limited cycle lifetime [24, 25]. Due to the persistent challenges associated with the rechargeability of AABs, research focus has shifted from rechargeable to primary AAB configurations, which are technically more suitable for Power‐to‐X applications. Although other rechargeable metal–air systems such as Zn‐air and Mg‐air exist, they are less suited for long‐term, large‐scale energy storage due to lower energy density and limited industrial integration. Primary AABs are therefore considered as promising alternatives for LTES applications [26]. The challenges associated with recovering the substantial heat released during direct combustion and steam oxidation, combined with the techno‐economic complexities of hydrogen handling, have motivated the exploration of electrochemical systems that enable the direct conversion of aluminum into electricity. AABs utilize the oxidation of aluminum at the anode and oxygen reduction at the air cathode to produce electricity with high theoretical energy density (8.1 kWh kg^−1^ Al) and without the need for external hydrogen handling [11, 27]. These characteristics make AABs strong candidates for stationary LTES applications, where almost zero self‐discharge, long‐term storability, and independence from pressurized gases or cryogenic systems are key advantages over conventional hydrogen‐based storage [28]. Recent progress has been reported for primary AABs, achieving aluminum conversion efficiencies exceeding 95% with negligible hydrogen evolution by optimizing the anode‐cathode configuration [11, 29]. Despite these promising developments, the environmental sustainability of AAB systems remains largely unexplored, particularly under large‐scale deployment scenarios. To the best of the author's knowledge, no previous study has systematically evaluated the life cycle environmental performance of AAB‐based LTES systems or compared them to established energy carriers.

To fill this gap, the present study performs an exploratory life cycle assessment (LCA) of a large‐scale LTES system employing primary AABs. The assessment seeks to identify key environmental hot spots to support the early‐stage design and sustainability optimization of this emerging technology. Two supply pathways are examined to ensure contextual robustness in comparison with gaseous hydrogen (GH_2_) stored in an underground storage and liquid hydrogen (LH_2_): (i) domestically produced aluminum in Germany and (ii) aluminum partly (30%) imported from Saudi Arabia. The inclusion of Saudi Arabia is motivated by its ambitious national strategy to expand large‐scale green hydrogen production, supported by vast renewable energy resources [30]. Therefore, Saudi Arabia provides a representative context for assessing the environmental impacts associated with H_2_ imports in LTES context, serving as a realistic benchmark for comparison with AAB‐based systems rather than reflecting the country's aluminum production relevance. Accordingly, the present study provides an anticipatory and comparative environmental assessment of primary AAB‐ and H_2_‐based LTES systems (both underground and LH_2_ storage) under given supply chain considerations. The outcomes contribute to the ongoing discourse on metal‐based energy carriers by identifying the environmental trade‐offs and design priorities that could shape their future role in global energy transition strategies.

## 2 Methodology

This chapter outlines the methodological approach employed for the LCA framework, considering system boundaries, data sources, impact assessment methods, and scenarios.

### 2.1 LCA Framework

The environmental performance of the AAB‐based LTES system was evaluated using the LCA methodology in accordance with the international standards ISO 14040 and ISO 14044 [31]. The LCA provides a systematic framework for quantifying the environmental impacts of products and processes across their entire life cycle, encompassing raw material extraction (‘cradle’) through production, use, and end‐of‐life treatment (‘grave’). The methodological procedure follows the four iterative stages of the ISO framework: (1) goal and scope definition, (2) life cycle inventory (LCI) analysis, (3) LCI assessment (LCIA), and (4) interpretation of results.

This study aimed to conduct a comparative LCA to evaluate the environmental sustainability of primary AABs for LTES applications, in comparison with two H_2_‐based reference systems. The system boundaries are defined as cradle‐to‐use, encompassing raw material extraction, production, assembly, and operation. Since this is an exploratory study, the primary aim is to identify the total impacts associated with the use of AAB in a LTES. Hence, the functional unit is defined as 1 kWh of stored electricity without allocation on coproducts hydrogen and heat.

**FIGURE 1:**
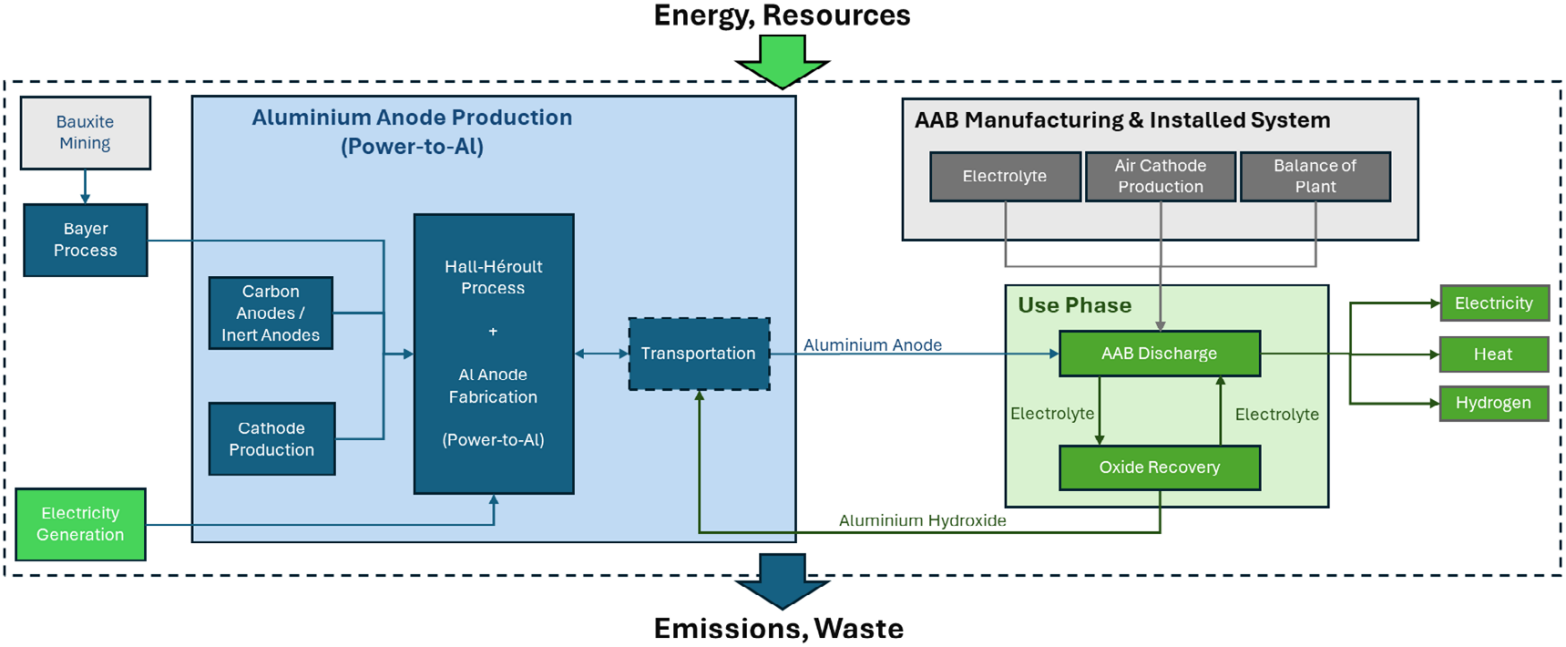
System boundaries of the AAB LTES system illustrating the interconnected processes of aluminum anode production, AAB manufacturing and operation, and Al_2_O_3_ recovery within a closed‐loop power‐to‐aluminum framework.

The defined system boundary encompasses the manufacture of AAB LTES system components, fabricated aluminum anodes, and the use phase, while excluding system dismantling and end‐of‐life treatment processes. Nevertheless, since aluminum serves as an energy carrier in the LTES system, its inherent circularity (i.e., aluminum is oxidized during discharge and subsequently regenerated from aluminum oxide (Al_2_O_3_)) reflects a cradle‐to‐grave concept within the system's operational loop, representing a closed‐loop material cycle. The cradle‐to‐use system boundaries are illustrated in Figure 1, introducing the modeled system and its three main subsystems:

1. **Aluminum production (Power‐to‐Al)**: The upstream processes of bauxite mining, Al_2_O_3_ extraction via the Bayer process, and primary aluminum production through the HH process are included in this subsystem. Also accounting for carbon or inert anode fabrication, cathode production, and power‐to‐Al pathways. The aluminum produced in this stage is processed through ingot casting and sheet rolling processes to fabricated anode sheets used in the AAB cells.
2. **AAB manufacture and installed system**: This subsystem covers the production and assembly of all major AAB components, including the electrolyte preparation (KOH and additives), air cathode fabrication, and balance‐of‐plant (BoP) elements.
3. **Use phase**: The operational phase includes the AAB discharge process, during which aluminum anodes are oxidized to produce electricity, heat, and minor quantities of H_2_. Post‐discharge, the electrolyte undergoes Al(OH)_3_ recovery, with the separated oxides transported back to the HH process for regeneration to Al_2_O_3_ and conversion into secondary aluminum anodes.

**FIGURE 2:**
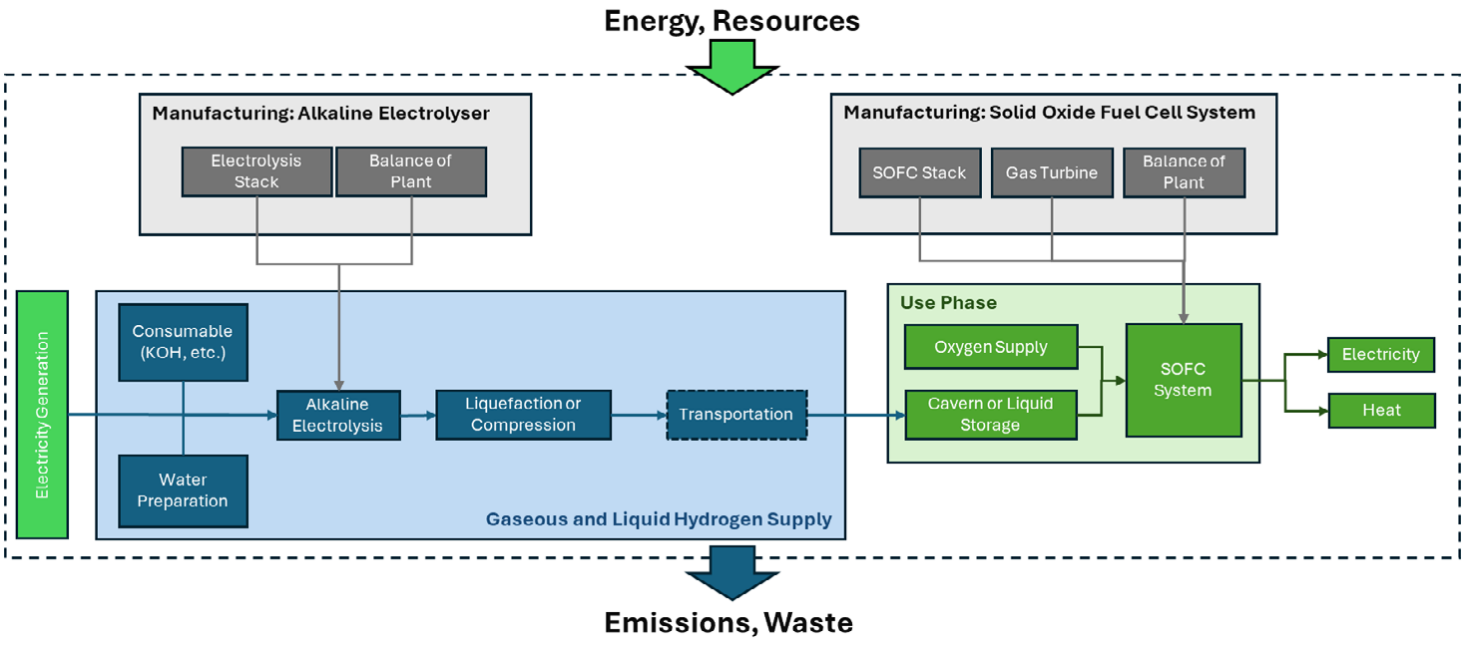
System boundaries of the reference H_2_ energy storage system, encompassing alkaline electrolysis, H_2_ compression/liquefaction, cryogenic/underground storage, and reconversion via a SOFC‐GT system.

To establish a consistent benchmark for the conducted assessment, two reference H_2_‐based LTES systems (one with GH_2_ stored locally in an underground storage and the other with LH_2_ storage) are introduced for comparison under analogous functional conditions. As illustrated in Figure 2, identically the system boundaries for H_2_‐based LTES cover cradle‐to‐use phases. The manufacturing stage encompasses the production of both upstream (H_2_ production) and downstream (H_2_ storage and utilization) subsystems. On the upstream, the alkaline electrolysis cell (AEC) system includes the manufacture of electrolysis stacks and BoP components. The downstream conversion infrastructure accounts for the fabrication of solid oxide fuel cell (SOFC) stacks, gas turbine (GT) unit, as well as auxiliary BoP components constituting the SOFC‐GT unit. In the use phase, hydrogen produced via water electrolysis is either compressed or liquefied (depending on the scenario), stored, and subsequently supplied to the SOFC‐GT system for electricity and heat generation. Identically, the functional unit for the reference system is selected as 1 kWh of stored electricity without allocation.

This configuration defines a fully integrated H_2_ storage chain, the GH_2_ stored in underground providing a representative and technologically mature counterpart to the AAB concept with to evaluate efficiency, energy density, and environmental performance within a harmonized comparative framework extended with LH_2_ considering the geographical limits associated with storage location and imports. Further elaboration of the system definition of both AAB and H_2_ systems are provided in the subsequent sections.

The LCIs were developed using a combination of primary and secondary data sources. For the AAB system, primary data were collected from laboratory‐scale prototype experiments, while secondary data were obtained from the ecoinvent v3.11 database and relevant literature for supporting processes. The complete LCI is provided in the Supporting Information (SI) to ensure transparency and reproducibility. For both LH_2_ and GH_2_ systems, secondary data from literature and ecoinvent v3.11 were used to model hydrogen production, compression, liquefaction, storage, and utilization.

The LCA models for AAB and reference systems were developed and simulated in the OpenLCA software environment using ecoinvent v3.11 for background data. Environmental impacts were assessed according to the Environmental Footprint (EF) 3.1 LCIA method, recommended by the European Commission and widely applied in battery and energy storage LCAs. A relative impact comparison was performed across all 16 impact categories defined in the EF 3.1 method. Based on their relative significance and the observed differences between the AAB and H_2_ systems, four key categories were selected for detailed discussion in this manuscript: acidification (AE), global warming potential (GWP_100_), eutrophication (EP: freshwater), and abiotic depletion potential (ADP: elements). Results for all 16 impact categories are provided in the SI.

### 2.2 Conceptual Design: Primary AAB System

**FIGURE 3:**
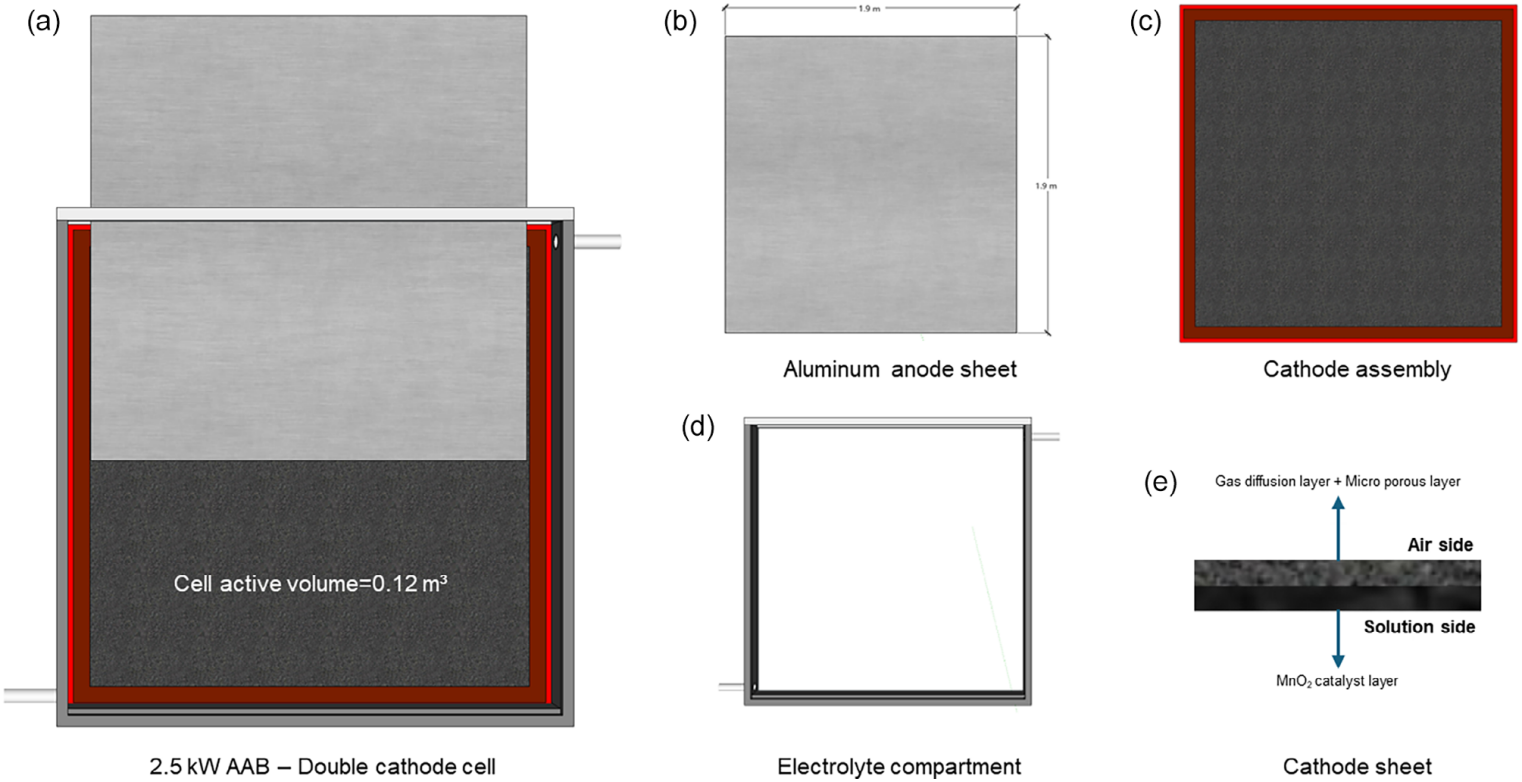
Primary AAB cell design.

The laboratory‐scale demonstration prototype developed at the Karlsruhe Institute of Technology served as the basis for modeling the primary AAB system. A 2.5 kW primary AAB cell was designed as the elementary module for large‐scale implementation (Figure 3 illustrates its counterparts). Upscaling from the laboratory‐scale prototype to the 2.5 kW module was based on expert consultations and exchanges with technology developers to ensure that scale effects were adequately reflected in the design while maintaining realistic performance expectations. A computer‐aided design (CAD)‐based cell design was developed using the specifications defined through the expert interactions to approximate a technically and operationally feasible large‐scale configuration. As illustrated in Figure 3a, the developed primary AAB cell features a modular configuration with overall dimensions of 2.2 × 2.2 × 0.2 m, accommodating an active cell volume of 0.12 m^3^. As an electrochemical energy conversion device, the AAB requires both a positive and a negative electrode. Although separators in batteries are generally employed to maintain physical separation between electrodes while permitting ionic conduction, this design excludes a separator to avoid mass transport limitations and performance losses identified by the developers. The cell assembly comprises a mechanically fed aluminum anode and dual air cathodes mounted on both sides to facilitate efficient oxygen reduction reactions (ORRs).

The air cathode exhibits a dual‐layer structure, consisting of an *air‐side* and a *solution side*, to promote optimal gas diffusion and electrochemical activity at the electrode–electrolyte boundary. Additionally, the air‐side cathode assembly incorporates air channels that enable continuous airflow, ensuring a consistent oxygen supply to the reaction surface. The electrolyte compartment, shown in Figure 3d, contains a potassium hydroxide (KOH)‐based alkaline electrolyte circulated through an external loop during discharge. The electrolyte is introduced into the cell only during operation and stored separately in designated tanks during standby. During discharge, the aluminum anode is oxidized, initially forming potassium aluminate (KAl(OH)_4_), which is subsequently converted to aluminum hydroxide (Al(OH)_3_) during a 24‐h resting period in the used‐electrolyte tank facilitated by specific additives as described in Section 2.2.3 [29]. The resulting Al(OH)_3_ can then be thermally processed, for example, using waste heat from the smelter facility to produce Al_2_O_3_ suitable for direct reuse in aluminum production [32]. Meanwhile, the regenerated electrolyte is filtered and returned to the system, thereby establishing a circular operational loop without additional oxide processing. The subsequent sections provide a detailed description of the cell components and the overall system architecture.

#### 2.2.1 Aluminum Anode

The aluminum anode acts as an energy storage media in a primary AAB. Since it is a mechanically rechargeable battery, aluminum sheets are stored and fed into the cell during operation through a feed system. Each anode sheet provides the large active aluminum surface required for electrochemical oxidation in contact with the alkaline electrolyte. Based on system design requirements, a nominal cell voltage of 1.5 V and a specific energy conversion capacity of 4.29 kWh_e_ kg^−1^ of aluminum necessitate an active electrode surface area of 3.6 m^2^ to achieve a cell output of 2.5 kW (see Figure 3b). The cell operates at a current of ≈ 1739 A, signifying moderate current densities that make the configuration suitable for high‐energy applications. Accordingly, the anode sheets are designed as 1.9 m wide, with a thickness of ≈0.5 mm, and a thickness reduction rate of 0.0165 μm s^−1^ under steady operation. The strong alkalinity of the KOH electrolyte ensures immediate activation of the aluminum surface upon contact, sustaining the oxidation process during discharge.

The mechanical feeding assembly was not included in the present modeling framework, as its final design and maintenance concept have not yet been defined. Due to this uncertainty, it was not possible to make reliable assumptions regarding its material composition, energy demand, or operational behavior. Qualitatively, such a subsystem is expected to consist primarily of structural elements, and a small electric drive. Given its comparatively low material intensity and long service lifetime relative to aluminum anode, its environmental contribution is expected to be minor. Nevertheless, once the design specifications are finalized, the component can be integrated into future model updates for completeness.

#### 2.2.2 Air Cathode Assembly

The air cathode enables the ORR, which sustains the overall electrochemical process. The cathode assembly consists of a three‐layer cathode sheet, air channels positioned on the air‐facing side, and structural supports designed for mounting on both sides of the cell wall, as shown in Figure 3c. The cathode sheet is structured sequentially from the air side to the electrolyte side, comprising a gas diffusion layer (GDL), a microporous layer (MPL), and a catalyst layer (see Figure 3e). For the ORR, the developers have proposed *α*‐MnO_2_ catalysts as a more environmentally friendly and cost‐effective alternative to conventional Pt/C cathodes, although other prominent cathode materials have also been reported for AAB configurations [33, 34, 35]. The complete cathode sheet exhibits an areal density of ≈5 kg m^−2^, requiring an active surface area of 3.6 m^2^ on each side of the cell to maintain design symmetry with the aluminum anode. This configuration corresponds to a total cathode mass of 36 kg per cell. The current collector for the air cathode was not explicitly included in the present analysis due to strong focus on the active ORR demonstration related to the TRL of the primary AAB. At this stage, the system is evaluated at a proof‐of‐concept and exploration level, where the focus is placed mainly on the electrochemical behavior of the electrodes rather than on full cell integration. Recent work by Rampf et al. (2026) indicates that the use of Ni‐mesh current collectors is particularly effective for enhancing overall efficiency when paired with the assessed air cathode [36]. In the present configuration, direct electrical contact from the cathode is assumed, thereby excluding the incorporation of an additional metallic or carbon‐based current collector to complete the circuit. The designed cathodes facilitate efficient oxygen diffusion through the air channels and ensures its subsequent electrochemical reduction, with the resulting oxygen species transferred through the electrolyte to support the oxidation of the aluminum anode.

#### 2.2.3 Alkaline Electrolyte

The alkaline electrolyte serves as a multifunctional component within the AAB system. In addition to its primary role as an ionic conductor, it performs two critical system functions: internal temperature regulation and removal of reaction products (converted oxides) from the cell. For effective thermal management, the electrolyte flow rate is controlled to ensure adequate heat exchange and heat removal, with the recovered heat transferred through an integrated heat exchanger to enhance overall system efficiency. The cell operating temperature is maintained at 20°C, as specified by the system developers, while the electrolyte outlet temperature can reach 80°C under nominal operation. During discharge, the oxidation of 1 kg of aluminum releases ≈3.7 kWh_th_ of heat, which can be recovered for waste heat utilization, thereby contributing to multifunctional system performance. To maintain the desired temperature balance, the required electrolyte circulation rate is estimated at 3.95 kg s^−1^, corresponding to about 97 m^3^ of electrolyte per 8‐h discharge cycle. To enable continuous operation of the AAB system, an electrolyte management strategy was developed based on a three‐stage electrolyte cycle comprising discharge, resting, and regeneration phases. After 8 h of daily operation, the used electrolyte is isolated for 24 h to allow Al(OH)_3_ precipitation and further conversion to Al_2_O_3_. The regenerated electrolyte is subsequently filtered, chemically adjusted, and reintroduced into the fresh tank. The entire process is managed via an automated valve and control system that regulates flow, temperature, and chemical composition, ensuring efficient electrolyte recovery, heat utilization, and material circularity.

#### 2.2.4 Primary AAB LTES

Describing the 2.5 kW cell unit and its associated components provides the foundation above for introducing the overall system design, which delivers a total power output of 1 MW. The large‐scale LTES system is structured hierarchically to ensure scalability and operational efficiency. As summarized in Table 1, the system achieves a nominal design power of 1 MW and an energy‐to‐power ratio of 8, corresponding to a total energy capacity of 8.3 MWh. The full system comprises 400 individual cells connected in series to increase the system voltage.

**TABLE 1:** Summary of key design and operational parameters of the 1 MW AAB system.

	Amount	Unit
Design power (design, actual)	1000, 1043	kW
Energy capacity (design, actual)	8000, 8344	kWh
Heat rate^a^	860, 774 @ *η*_heat exchanger_ = 90%	kW
Parasitic H_2_ evolution rate	1.3	kg h^−1^
Energy power ratio	8	–
Aluminum feed rate	≈233	kg h^−1^
Total electrolyte inventory	390	m^3^
Electrical efficiency	53%	
Thermal efficiency	41%, 37% @ *η*_heat exchanger_ = 90%	
Total efficiency	89%	
RTE electrical^b^	32%	
RTE total^b^	54%	

a Heat rate in combined heat and power (CHP) mode.

b Assuming an aluminum production electricity intensity of 13.4 kWh kg^−1^.

The aluminum feed rate required to sustain continuous operation is 0.0648 kg s^−1^, enabling an electrical conversion efficiency of 53% and a thermal efficiency of 41%, resulting in an overall total efficiency of ≈89% based on the upscaled system performance. When considering the regeneration and reuse of reaction products, the electrical RTE reaches 32%, and the overall RTE goes up to 54% if heat recovery is taken into consideration. The system releases ≈860 kW (ca 6.2 MWh_th_ at 90% heat exchanger efficiency) of recoverable heat during 8‐h operation, which can be harnessed through the electrolyte circulation loop for district heating or waste heat recovery. Although the heat recovery system is included in the model through the consideration of a heat exchanger, the downstream utilization of the recovered heat is not modeled. Hence, the assessed environmental impacts are calculated without allocation among heat, hydrogen, and electricity, referring to electricity as the sole functional output in accordance with the defined goal and scope of the study.

**FIGURE 4:**
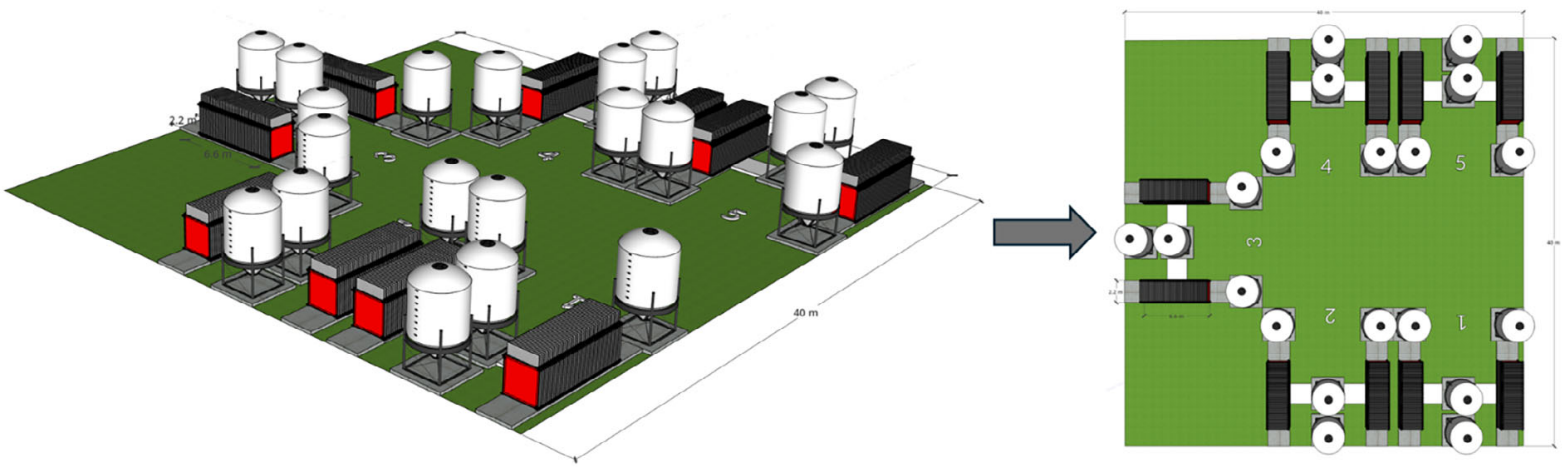
Schematic representation of the AAB system architecture from cell to full‐scale integration.

As illustrated in Figure 4, unit AAB cells are first assembled into 100 kW stack modules, with each module consisting of 40 interconnected cells. These stack modules are subsequently integrated to form the full‐scale 1 MW AAB installation, comprising five 200 kW operational units. The three‐dimensional layout (left) shows the physical arrangement of the stack modules and the adjacent electrolyte tanks. Each 200‐kW unit includes two 40‐cell stack modules and is equipped with four cylindrical electrolyte tanks each accommodating ca. 22 m^3^ of electrolyte. The tanks are designated for fresh electrolyte supply, used electrolyte collection, and electrolyte regeneration in accordance with the defined electrolyte management strategy. The schematic plan view (Figure 4, right) identifies the five groups (labeled 1–5) distributed over a 1600 m^2^ platform.

This arrangement minimizes piping length, reduces hydraulic losses, and ensures balanced electrical and thermal distribution across the installation while providing central clearance for auxiliary equipment and maintenance access. To enable stable and autonomous operation, the system integrates essential BoP components, including pumps, heat exchangers, piping and cabling systems, a power conversion and inverter unit, and automated control systems. Installation‐related efforts are not considered, as the assessed system is at a conceptual design stage and reliable estimates are not yet feasible. This modular and scalable architecture enables efficient electrolyte circulation and oxide regeneration establishing the AAB LTES system.

### 2.3 Conceptual Design: GH_2_‐ and LH_2_‐Based LTES

For the reference H_2_ system, the water electrolysis model follows the framework established by Wei et al. (2024), incorporating configuration and performance data from Gerloff (2021) and manufacturer specifications from IndWEDe project data [37, 38, 39]. The electrolyzer stack is characterized by a lifetime of 55,000 operating hours, requiring three stack replacements over a 27.5‐year total system lifetime. The AEC unit operates at a temperature range of 60–80°C and a pressure of ≈20 bar, consuming 51.8 kWh kg^−1^ H_2_ of electricity at 64.5% efficiency. The system produces 19.36 kg H_2_  h^−1^, and at an annual operating time of 8000 h, the cumulative H_2_ output over the system's lifetime amounts to ≈4.26 million kg H_2_.

Following electrolysis, the produced hydrogen is either conveyed via pipelines to underground storage or converted into liquid form to enhance volumetric energy density according to the considered pathway. The underground storage system was modeled based on the Hystories EU project [40]. For the LH_2_ supply, the produced GH_2_ is cryogenically liquefied and transported to a storage unit (either underground storage upon regasification or LH_2_ storage tank), and subsequently supplied to an SOFC system integrated with a GT for heat recovery. The liquefaction of gaseous hydrogen is estimated to require between 8 and 12 kWh kg^−1^ H_2_, consistent with industrial‐scale averages for low‐temperature compression and refrigeration processes. Hence, assuming an average value of 10 kWh kg^−1^ H_2_ is considered representative [41]. In addition, process‐related H_2_ losses are incorporated in the system model to better reflect operational realities. The baseline case presented in this study is established on the input of experts and therefore regarded as representative of practical conditions. The indicative loss rates summarized in SI are applied in the sensitivity analysis for completeness. The supply chain losses assumptions are subsequently linked to the downstream use of H_2_ in the SOFC‐GT system for re‐electrification.

The SOFC‐GT system design proposed by Primas & Hoffmann (2007) for natural gas operation was adapted to reflect the relevant modifications for the present application and subsequently implemented in the model as described in the LCI [42]. The performance characteristics and fuel requirements of the H_2_‐based SOFC system are summarized in Table 2. The complete conversion unit is designed for a nominal capacity of 1 MW, operating at 58% electrical efficiency and 80% total efficiency. These efficiencies correspond to the cogeneration of electricity and recoverable heat at 80% hydrogen utilization ratio.

**TABLE 2:** Summary of key design and operational parameters of the LH_2_ system.

	Amount	Unit
Design power (SOFC, GT)	1000 (695, 305)	kW
Annual full load hours	2880	h a^−1^
Heat rate	≈625	kW
Hydrogen flow rate	≈65	kg h^−1^
Hydrogen utilization ratio	80%	
System lifetime	20	years
Electrical efficiency^a^	58% (46%, net)	
Total efficiency^a^	80% (75%, net)	
RTE	30% (27%, net with underground storage)	

*Note:* Values in brackets indicate net efficiencies, accounting for supply losses.

a Performance data based on Primas & Hoffmann (2007) [42].

The system has an overall lifetime of 67,360 operating hours, with stack and turbine replacements accounted for based on individual component service lives of 48,000 h for the SOFC and 50,000 h for the GT. The system function considered here is only the electricity storage identical to the AAB system without allocation. The hourly H_2_ demand is estimated at ≈65 kg h^−1^ for the named power capacity. Overall, the combination of H_2_ production and SOFC‐GT utilization define a complete power‐to‐hydrogen‐to‐power configuration. This integrated pathway provides a benchmark for comparison with the AAB system in terms of RTE (30%), thermal energy recovery, and life cycle system performance.

### 2.4 Use Case: LTES Application

**FIGURE 5:**
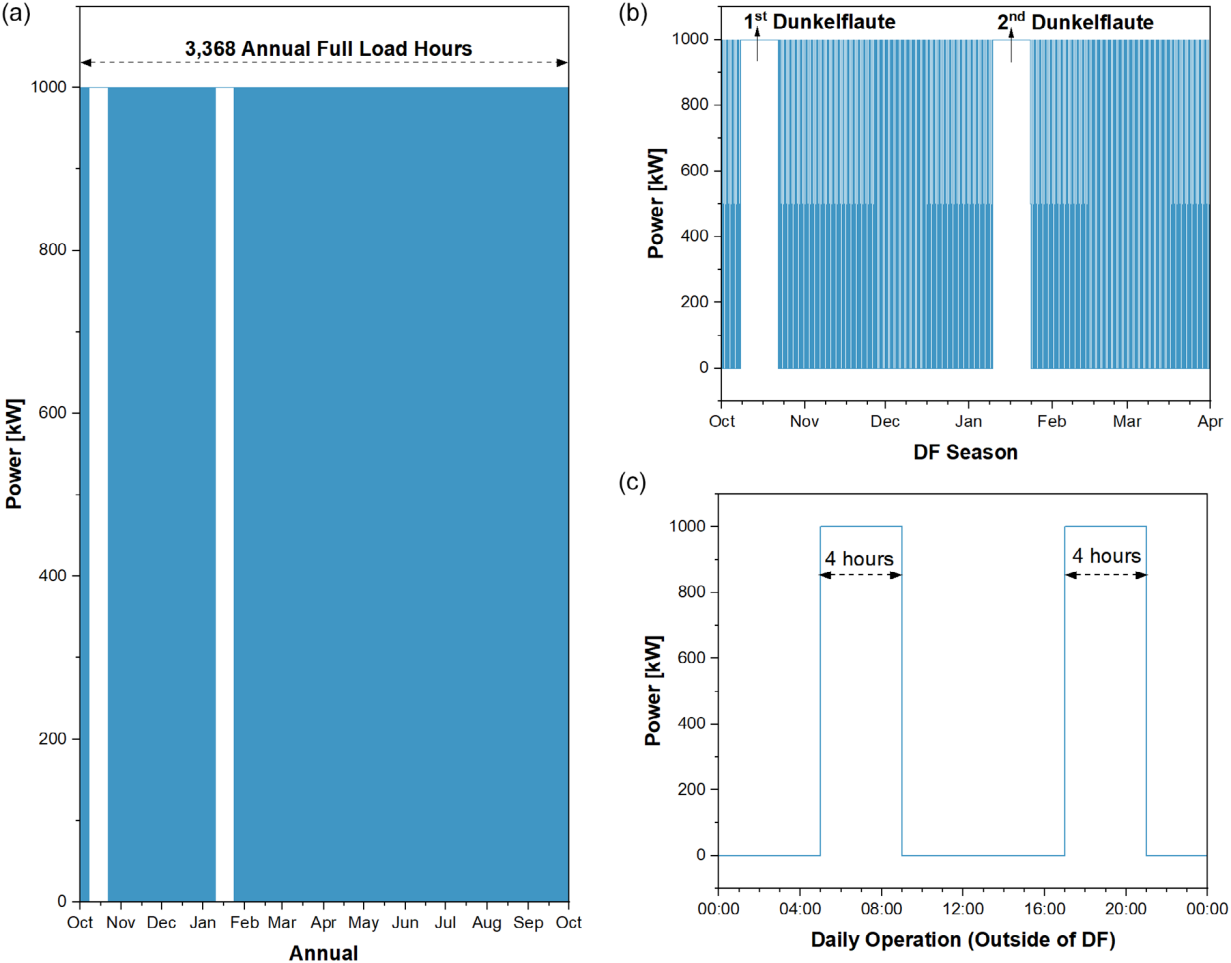
Operation schedule of the LTES system.

The LTES application is designed to provide the required storage capacity throughout the year by supplying electricity to the grid daily 8 h, split into two 4‐h blocks (05:00–09:00 and 17:00–21:00, see Figure 5). Additionally, annual operation includes long‐duration Dunkelflaute (DF) events anticipated in October and January, during which the system operates continuously for 24 h over 14 consecutive days, twice per year, to cover sudden drops in wind and photovoltaic (PV) generation.

In total, this amounts to 3368 full‐load hours per year (≈38% capacity factor). For both AAB and H_2_ (gaseous and liquid) storage systems, the long‐term storage is exercised under this energy storage scenario, with storage mainly deployed outside the DF period (April–October).

### 2.5 Scenarios

Four scenarios are defined to assess the life cycle environmental performance of the AAB LTES, focusing on the influence of smelting technologies, electricity sources, and supply‐chain configurations. Since aluminum production represents the ‘charging’ phase of the AAB storage cycle, the sustainability of the overall system is closely linked to the environmental characteristics of the smelting process. Table 3 summarizes the configuration of each scenario, highlighting key differences in the smelting technology, electricity and heat supply, and production location. The AAB‐conventional scenario reflects current industrial practice based on the conventional HH process employing carbon anodes, powered by German grid electricity (2023), and using natural‐gas‐based heat supply. This configuration serves as the reference case for present‐day aluminum production, characterized by substantial GHG emissions from carbon‐anode oxidation during electrolysis (forming CO, CO_2_, and PFCs). State‐of‐the‐art scenario (AAB‐SoA) maintains the carbon‐anode HH configuration but replaces the grid mix with a renewable‐based electricity supply (70% wind:30% solar) while utilizing natural gas in a SOFC‐GT system for heat supply. For AAB‐SoA, efficiency gains are attributed to process optimization, reduced anode‐cathode distance, and enhanced thermal management, yielding 12.0 kWh kg^−1^ Al [43]. This scenario represents an intermediate, partially decarbonized pathway through integration of renewables and efficient heat conversion.

**TABLE 3:** Summary of aluminum production pathway scenarios and assumptions.

Scenario	On‐site			30% Import
	AAB‐conventional	AAB‐SoA	AAB‐inert	AAB‐inert
Anode type	Carbon	Carbon	Inert anodes	Inert anodes
Anode lifetime	25 days	25 days	2 years	2 years
Electricity	German grid, 2023	70:30%, Wind:Solar	70:30%, Wind:Solar	30:70%, Wind:Solar
Aluminum production location	Germany	Germany	Germany	30% imported from Saudi Arabia
Heat source	Natural gas	Natural gas in SOFC	Natural gas in SOFC	Natural gas in SOFC
Smelting intensity [kWh kg^−1^ Al]	13.4^a^	12^b^	14.9^c^	14.9^c^

a World average aluminum smelting intensity.

b State‐of‐the‐art smelters energy intensity.

c Average electricity intensity for inert anodes.

AAB‐inert (on‐site) scenario introduces inert anodes combined with the same renewable electricity mix (70% wind: 30% solar) and SOFC‐based natural‐gas heat supply. Although this configuration eliminates direct process emissions from carbon oxidation, it entails higher energy intensity due to increased electrochemical potential and thermal losses, modeled at 14.9 kWh kg^−1^ Al [43]. AAB‐inert (import) scenario replicates the on‐site inert scenario to import configuration where 30% of the aluminum anodes are produced with inert‐anode smelting located in Saudi Arabia using a renewable electricity mix (30% wind: 70% solar) and SOFC‐based heat supply. The aluminum anodes are transported to the storage site in Germany, and the discharged aluminum oxides are returned for regeneration. The presented scenarios account for regional variations in carbon intensity across the considered energy mixes and incorporate process differences within the constraints of available data, providing a broader geographical perspective. The performed sensitivity analysis evaluates the effects of varying regional electricity sources and aluminum production carbon intensities on the environmental outcomes as presented in Section 3.4.

To ensure methodological consistency across systems, the comparison is carried out considering identical geographical and energy supply conditions for inert on‐site and import scenarios for the GH_2_ and LH_2_ supply chains, thereby allowing direct comparison of the two LTES concepts as presented in Table 4.

**TABLE 4:** Summary of hydrogen production pathway scenarios and assumptions.

Scenario	On‐site		30% Import	
	**GH**_**2**_	**LH**_**2**_	**GH**_**2**_	**LH**_**2**_
Storage type	Underground storage	Cryogenic LH_2_ tank	Underground storage	Cryogenic LH_2_ tank
Transportation	Pipeline	Maritime and land transport	Pipeline, maritime, and land transport (LH_2_)	Maritime and land transport
LH_2_ share in overall supply	0%	100%	30%	30%
Production location	Germany	Germany	Germany	Saudi Arabia
Electricity	German grid, 2023	70:30%, Wind:Solar	70:30%, Wind:Solar	30:70%, Wind:Solar
Electrolysis intensity [kWh kg^−1^ GH_2_]	51.8			

Similar to the aluminum supply chain, the reference H_2_ system evaluates both GH_2_ and LH_2_ supply under on‐site production and import scenarios for LTES. For GH_2_, locally produced hydrogen is transmitted via a pipeline to an underground storage facility and then consumed in the SOFC‐GT system colocated with the underground storage, representing the most favorable LTES configuration. To address the geographical limitations of underground storage, an alternative LH_2_ option with a liquid‐tank storage system is also considered where hydrogen from the same source is liquefied and stored in an on‐site cryogenic LH_2_ storage tank. In the import scenarios, 30% of hydrogen is sourced from Saudi Arabia. In both GH_2_ and LH_2_ import cases, hydrogen is transported in liquid form; on‐site, GH_2_ is stored as gaseous hydrogen in the underground storage upon regasification, whereas LH_2_ is stored as liquid hydrogen in a cryogenic storage tank.

### 2.6 Life Cycle Inventory Modeling

In this study, detailed LCIs are developed for the electrochemically active components of the AAB system, namely, the anode, cathode, and electrolyte, as these subsystems govern the overall electrochemical performance and material exchange behavior. The modeling is based on experimentally validated data and scaled to reflect large‐scale operational conditions, as detailed in the following subsections.

The remaining BoP components, including the stack housing, tanks, circulation pumps, piping, heat exchangers, and power‐conditioning units, are modeled using Weber et al. (2018) and ecoinvent v3.11 [44]. This approach is justified by the structural and functional similarities between the AAB and vanadium redox flow battery (VRFB) systems, both featuring modular stacks, electrolyte circulation, and comparable auxiliary infrastructure. The BoP components are therefore not discussed in detail here; full inventories and modeling parameters are provided in the SI to ensure transparency and reproducibility. An overview of the integrated 1 MW AAB system inventory is presented in Table 5, summarizing the principal flows within the system‐level LCI.

**TABLE 5:** Life cycle inventory of AAB battery energy storage system. Reference unit: AAB System, 1 MW.

	Flow	Amount	Unit
Input	Cathode fabrication	2882	m^2^
	KOH electrolyte, with additives	389	m^3^
	Cable, copper	35,400	kg
	Pipes	15,177	kg
	Electrolyte tank, for AAB	46,953	kg
	FKM gasket	3076	kg
	Heat exchanger	1023	kg
	Inverter production, 500 kW	2	Item(s)
	Process control system	13	kg
	Pump	1040	kg
	Stack frame, steel	2316	kg
	Cell housing, PVC	221,127	kg
Output	AAB System, 1 MW	1	Item(s)

#### 2.6.1 Aluminum Anode

The aluminum anodes are modeled as produced from aluminum derived via the HH process, followed by a sheet rolling step upon ingot casting to manufacture the anode rolls in the required dimensions for integration into the AAB system. The aluminum anodes are modeled considering the International Aluminium Institute (IAI) inventories provided in ecoinvent v3.11. Regional adjustments are applied to the scenarios in Section 2.5 for electricity intensity and process efficiency. As the functional unit is defined as 1 kWh of stored electricity, the modeling accounts for the net energy input required for the storage cycle. For instance, in the AAB‐conventional scenario, aluminum production via the conventional HH process requires 13.4 kWh kg^−1^ Al of electricity, while the subsequent discharge of 1 kg of Al in the AAB system releases 4.29 kWh. Accordingly, the net energy consumption for the baseline aluminum‐based storage cycle is determined as 9.11 kWh kg^−1^ Al.

As no dedicated public LCI dataset currently exists for metallic inert anodes, a proxy process was developed to approximate their material composition and manufacturing requirements in inert‐anode scenarios. The LCI for the metallic inert anode was constructed using process from ecoinvent v3.11 based on the information available in the literature considering a ((Cu_65_Ni_20_Fe_15_)_98.6_O_1.4_) inert‐anode identified as the most promising laboratory‐ and pilot‐scale formulations [18, 43, 45]. Since industrial‐scale energy inputs for metallic inert‐anode fabrication are difficult to predict, the estimates are based on the laboratory procedures reported by Helle et al. and upscaled using a lab‐to‐market efficiency gain of 3–5× [46]. The lab protocol comprises high‐energy ball‐milling of Cu, Ni, and Fe powders under inert atmosphere, followed by sieving, cold‐pressing, and sintering at 900–1100°C. The laboratory‐scale energy use is estimated at 12.3 kWh kg^−1^ electricity and 11.5 MJ kg^−1^ heat which yields central values of ≈3.1 kWh kg^−1^ electricity (12.3/4) and ≈2.9 MJ kg^−1^ heat (11.5/4) for industrial scale. Accordingly, logarithmic normal distributions are defined to account for the uncertainties. For the inert‐anode configuration, the aluminum production inventory is accordingly modified to account for increased electricity intensity and the elimination of direct GHG emissions, reflecting the expected environmental performance improvements of this technology. Nevertheless, due to data limitations, wetted and drained cathodes are not modeled in the inert‐anode scenarios.

Following the inventory development for conventional and prospective smelting processes, the subsequent steps involve ingot casting and sheet rolling, which is required to fabricate aluminum anodes in the appropriate form for continuous feeding into the electrochemical cell. For this purpose, the existing ingot casting and sheet‐rolling processes available in ecoinvent v3.11 are adopted as the modeling basis and adapted to reflect the specific geometrical and processing requirements of the aluminum anodes.

For import scenario, the transport chain includes 400 km of inland road transport within Saudi Arabia, 10,000 km of maritime transport by bulk carrier, and 100 km of inland road transport within Germany using transport datasets in ecoinvent v3.11. In contrast, for the on‐site production scenarios, aluminum smelting and anode fabrication are assumed to take place in close proximity to the AAB installation, and therefore no transportation effort is considered.

Accordingly, primary aluminum is required only for the initial system charge, while in subsequent cycles, the recovered Al(OH)_3_ serves as the feedstock for electrolytic reconversion to metallic aluminum upon conversion to Al_2_O_3_. It is assumed that Al(OH)_3_ is recovered from the electrolyte with 100% efficiency and after conversion possesses smelting‐grade purity Al_2_O_3_, allowing its direct reintroduction into the HH process. Acknowledging that, this assumption represents an optimistic upper bound for system performance, any losses in recovery efficiency or degradation in purity would increase the demand for primary aluminum and its associated upstream impacts. However, because this assumption is applied continuously across all comparative scenarios, deviations from ideal recovery are not expected to alter the relative ranking of scenarios, but would primarily affect absolute impact values. Hence, the reintroduced Al_2_O_3_ is modeled without upstream environmental burdens, and only transport‐related impacts are considered where applicable in the respective scenarios as outlined within the system boundaries.

#### 2.6.2 Air Cathode Assembly

The air cathode LCI was developed by integrating empirical synthesis data for the α‐MnO_2_ catalyst, process‐level modeling of the Freudenberg H23C2 GDL, and subsequent cathode fabrication parameters. The α‐MnO_2_ catalyst inventory was constructed using laboratory‐scale synthesis data from Mainar et al. (2016), including the consumption of manganese(III) oxide, sulfuric acid, ethanol, and electricity for magnetic stirring and drying [47]. The total energy requirement for the laboratory‐scale synthesis of α‐MnO_2_ is estimated at 5.2 kWh kg^−1^. Applying a lab‐to‐market efficiency gain of 3–5×, the corresponding industrial‐scale electricity consumption is estimated at 1.28 kWh kg^−1^, with associated uncertainty represented by a 95% confidence interval. The process yields high‐purity α‐MnO_2_, while by‐products such as manganese sulfate and aqueous emissions are modeled according to stoichiometric balance and reaction efficiency. The GDL and MPL are modeled based on the commercial Freudenberg H23C2 material, in which the two layers are integrated to permit uniform catalyst deposition on the electrolyte‐facing surface. The GDL inventory was established based on the methodological framework and process data reported by Simons and Bauer (2015) and Evangelisti et al. (2017), further adapted to reflect the material composition and thickness specifications provided in the Freudenberg technical datasheet [48, 49]. The model accounts for the carbon fiber substrate, polytetrafluoroethylene (PTFE) binder, and polymeric backing materials, with energy and heat demands for lamination and thermal treatment represented through electricity and process steam inputs. All background processes were linked to ecoinvent v3.11, ensuring region‐specific electricity mixes and material markets representative of European manufacturing conditions.

The cathode fabrication process involves the integration of the GDL + MPL with the catalyst through a spray‐coating procedure, which consumes isopropyl alcohol and deionized water as solvents, along with electricity for spraying and drying operations. At the laboratory scale, this process exhibits significant catalyst ink losses due to overspray and deposition inefficiencies. To provide a conservative approximation of material utilization, a coating efficiency of 60% is assumed when estimating the required quantity necessary to achieve a catalyst loading of 0.3 mg cm^−2^. Considering the reported energy consumption range for ink‐spray coating processes of 1.5–3.5 kWh m^−2^, this value is adopted directly for an industrial‐scale cathode fabrication process [50]. Additionally, to ensure sealing integrity and electrolyte containment, 3 cm‐thick fluorocarbon elastomer (FKM) gaskets are used to mount the cathode assemblies securely on both sides of the cell.

#### 2.6.3 Alkaline Electrolyte

The electrolyte preparation process for the AAB system was modeled based on experimentally validated formulations and adapted for system‐scale implementation following Xu et al. (2023) [29]. The electrolyte consists of a 4 M KOH solution, prepared by dissolving solid KOH pellets in deionized water, followed by the addition of sodium stannate trihydrate (Na_2_SnO_3_·3H_2_O) to obtain the baseline ‘blank electrolyte’, requiring in total 390 m^3^ electrolyte. The electrolyte formulation incorporates Na_2_SnO_3_ and adipic acid as additives to enhance aluminum anode activation and improve Coulombic efficiency. Based on experimental data, the additive dosing rate is set at 10 mL min^−1^, which is upscaled accordingly. This composition and operational profile are incorporated into the LCI model to represent material flows, additive consumption, and electrolyte regeneration requirements.

All background processes including KOH, adipic acid and deionized water were linked to corresponding datasets in ecoinvent v3.11 under European market conditions. Since no dedicated dataset for Na_2_SnO_3_ exists in ecoinvent v3.11, its production was modeled according to the procedure described in the literature, assuming primary tin as the precursor material [51]. Based on this synthesis route, the energy consumption for Na_2_SnO_3_ is estimated to be 0.64 kWh kg^−1^.

#### 2.6.4 Reference System: LH_2_‐Power LTES

The LCI of the H_2_ reference system is modeled considering the literature data from Wei et al. (2024) based on Gerloff (2021), and Simoes et al. (2021) representing a state‐of‐the‐art AEC system [38, 39, 52]. Included are all BoP components necessary for continuous operation, comprising of the power electronics (rectifier, voltage adaptation units), control systems, H_2_ purification and drying units, water purification and feed systems, diaphragm compressor, gas separator, alkali‐resistant rotary pump, cooling and heat exchanger systems, and tanks for water, electrolyte, and H_2_ buffering. Additional infrastructure components such as piping, tubing, electrical cabling, structural containers, concrete foundations, and safety fittings are included within the system boundaries to ensure a comprehensive representation of the installed system. Material inputs, such as aluminum, stainless steel, copper, and polymeric parts are modeled using the corresponding ecoinvent v3.11 datasets under global market conditions for both, the stack and BoP components. Consistent with the AAB system modeling approach, the H_2_ scenarios also consider the net electricity consumption per kWh of stored electricity in the use case. Hence, the net stored electricity corresponding to the content of hydrogen (e.g., 14.27 kWh kg^−1^ H_2_ for on‐site supply and use) is deducted from the total process energy demand (51.8 kWh kg^−1^ H_2_ for electrolysis plus consumed energy for compression, liquefaction, and reliquefication other process steps).

The use phase model converts deionized water and KOH electrolyte into GH_2_ and oxygen coproduct (8 kg O_2_ per 1 kg H_2_). The oxygen produced during electrolysis is not allocated for further use or considered in the system's resource allocation. Produced hydrogen is stored either in an underground storage (as GH_2_) or in a cryogenic LH_2_ tank, depending on the scenario definitions. In on‐site scenario, GH_2_ is transported via a high‐pressure transmission pipeline (assumed length: 500 km) and subsequently through low‐pressure distribution lines (assumed length: 200 km) to the point of storage. The transmission and distribution infrastructure is modeled based on Tsiklios et al. (2022), including the placement of compressors at 125 km intervals to maintain pressure along both the high‐pressure transmission and low‐pressure distribution segments [53]. Underground storage LCI is modeled using the Hystories EU project Deliverable 6.3, which provides detailed inventories for construction and operation. These data underpin the representation of drilling, casing, leaching, surface facilities, and use phase inputs within the underground storage system [40]. For the GH_2_ storage import scenario, LH_2_ is delivered to the site to benefit from its higher volumetric energy density during transport. Upon arrival, the LH_2_ is regasified and injected into the underground, where it is stored in gaseous form. Prior utilization, the required GH_2_ is directed to an intermediate high‐pressure buffer tank which is modeled based on Wulf et al. (2018) [54].

In the alternative liquid‐storage scenario, imported LH_2_ remains in cryogenic tanks without regasification until needed. The LH_2_ supply chain includes maritime and inland transportation stages based on data from the Suiso Frontier pilot and projected commercial LH_2_ carriers [55]. The maritime transport leg is considered only for the import scenario, representing hydrogen delivery from Saudi Arabia to Germany over a distance of ≈10,000 km maritime in ca. 25 days. Both in the on‐site and import scenarios, identical inland transport distances are assumed of ≈400 km within Saudi Arabia and 100 km within Germany to ensure consistent treatment of local logistics and distribution impacts. For on‐site storage, the required tank capacity is estimated as 380 m^3^ with active re‐liquefaction to avoid boil‐off impacts. All background data for electricity generation, materials, and transport services are sourced from ecoinvent v3.11 under European or global market conditions.

The downstream utilization phase models the conversion of stored H_2_ into electricity and heat using an SOFC‐GT system. The LCI is based on the ecoinvent process ‘fuel cell, solid oxide, with micro GT, 180 kW_e_, future, electricity, low voltage’, adapted to reflect hydrogen as the sole fuel rather than natural gas [42]. This modification accounts for the thermodynamic and chemical properties of H_2_, including its lower heating value (LHV = 33.3 kWh kg^−1^) and lower volumetric energy density. Hydrogen input to the SOFC‐GT system is linked directly to the upstream H_2_ supply chain requiring 0.065 kg H_2_ per kWh_e_ releasing 0.6247 kWh_th_ per kWh_e_ heat at 80% hydrogen utilization ratio. All associated hydrogen losses through the supply chain and downstream utilization are incorporated into LCIs, as described in Section 2.3. The SOFC fuel utilization ratio is set at 80%, with the unreacted hydrogen after GT modeled as process loss. Oxygen supply is assumed at a stoichiometric ratio of 1.15 kg O_2_ per kWh electricity.

To accurately represent system performance, additionally PCS were incorporated to account for high‐efficiency DC/AC inversion and power management, supplementing the inverter already present in ecoinvent v3.11. Other BoP components are retained as modeled in the base process. Maintenance materials, lubricants, and waste mineral oil emissions are proportionally scaled to system capacity and lifetime. This configuration captures the full cradle‐to‐use phase of the LH_2_ energy storage system, encompassing hydrogen production, liquefaction, storage, transport, and downstream energy conversion to electricity and heat.

#### 2.6.5 Electricity Mix

The electricity supply mixes used in this study reflect both current domestic conditions and prospective renewable configurations relevant to the modeled scenarios. The composition of the electricity mixes applied for Germany (2023), and for the renewable‐based scenarios representing Germany and Saudi Arabia, are presented in (SI). The German (2023) mix corresponds to the average national grid composition for that year, with a renewable share of ≈53% integrating ca. 5% transmission and distribution network losses. For the on‐site renewables scenarios (SoA and inert), a prospective fully renewable mix is defined with a composition of 70% wind and 30% PV power. The Saudi Arabia renewables mix assumes an inverse distribution of renewable sources of 70% solar and 30% wind, reflecting the rapidly expanding PV infrastructure, complemented by wind resources. This configuration aligns with the reported initiatives, such as the NEOM project [56].

## 3 Impact Assessment Results

In this section, the cradle‐to‐gate impact assessment results of the AAB are presented. For the purpose of this assessment, ‘cradle‐to‐gate impacts’ are defined as the environmental burdens arising from the production and installation of the primary AAB system. Within the defined cradle‐to‐gate system boundaries, the environmental impacts associated with the aluminum anode are evaluated separately to provide a comprehensive overview of scenario‐specific effects. Subsequently, the analysis extends to the use‐phase impacts, beginning with a comparison between aluminum and H_2_ as energy carriers within the specific use‐case context, and culminating in the overall cradle‐to‐use impact assessment results with the inclusion of the use phase related‐impacts. It is important to note that all results are attributed exclusively to the electricity storage function of the system, without considering any allocation for by‐products such as heat or hydrogen. The assessment is further complemented by an uncertainty analysis performed through Monte Carlo simulation.

### 3.1 Cradle‐to‐Gate

**FIGURE 6:**
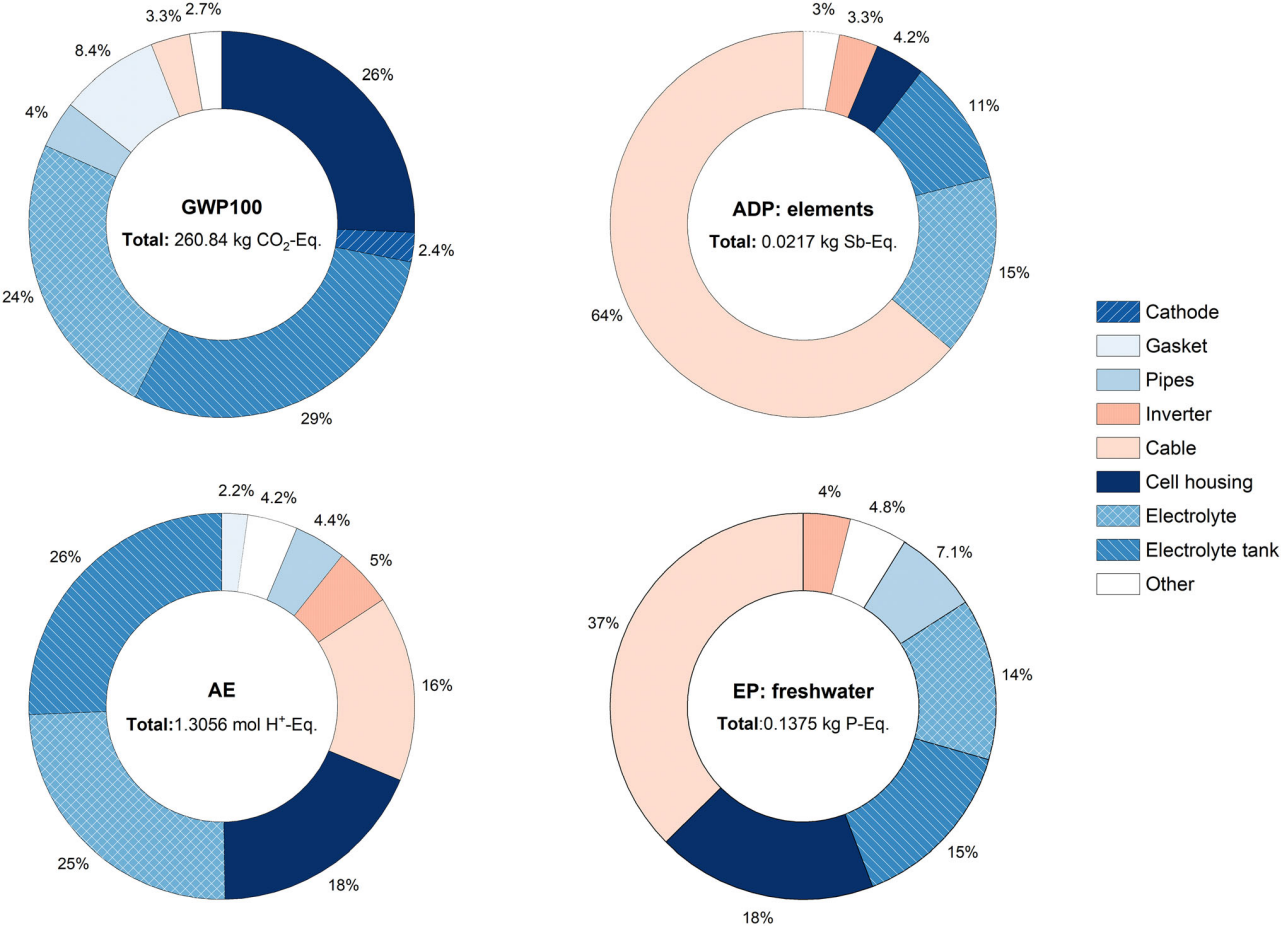
Manufacturing impacts of the AAB system on selected impact categories per kWh of installed electrical energy storage capacity.

The cradle‐to‐gate impacts presented first focus on the production‐related impacts of the AAB system, excluding the aluminum anode. This distinction is made because the aluminum anode does not constitute a static component of the AAB but is supplied to the system externally since the primary AAB is mechanically recharged. Figure 6, presents the impact assessment results of the AAB, considering both the active and the BoP components, across selected impact categories normalized per kWh of installed electrical energy storage capacity. As illustrated in Figure 6, no single component dominates all impact categories.

The total GWP_100_ impact distribution indicates that together the cell housing, electrolyte tank, and electrolyte account for nearly 80% of the manufacturing‐related impacts. These contributions predominantly originate from upstream processes, particularly the direct GHG emissions associated with polyvinyl chloride (PVC) production for the cell housing, xylene used in polymer resin manufacturing for the electrolyte tank, and the combined effects of KOH production and electricity consumption related to the electrolyte. Except for the pipes, gaskets, and cables, the remaining peripheral and BoP components contribute relatively minor to GWP impacts, each accounting for less than 2.7% of the total. On AE similar to GWP_100_, electrolyte tank and electrolyte exhibit the highest contributions, followed by the cell housing and cables due to upstream NO_*x*_ and SO_2_ emissions. These impacts are strongly linked to the production processes of polyester resin and glass fiber utilized in electrolyte tank manufacturing, and KOH employed in the electrolyte as well as PVC used in cell housing and copper use in cables.

In the ADP and EP categories, the dominance of copper cables is particularly notable, contributing 64% and 37% of the total impacts, respectively. Copper use is known to be characterized by high mineral resource consumption, and given its extensive use within the system, it constitutes the major contributor to ADP. The ADP impacts associated with cables are followed by those from the electrolyte and electrolyte tank, with the former influenced by the use of Na_2_SnO_3_·3H_2_O additives and the latter linked to glass fiber consumption required for corrosion‐resistant tank construction. Regarding EP impacts, the primary contributions from cables stem from emissions related to metal mining and chemical processing stages. Other major contributions originate from the cell housing (due to PVC production), the electrolyte (through KOH‐related processes), and the electrolyte tanks (through polyester resin and glass fiber production), all of which are associated with upstream phosphate emissions. Within the cradle‐to‐gate system boundaries, electrolyte, electrolyte tanks, cables, and cell housing are identified as the critical hotspots for improving the environmental performance of the AAB system in all impact categories.

Although the primary production impacts of aluminum used for anode fabrication contribute only marginally to the overall impacts, their relevance increases after the first discharge, when the HH process is integrated for reconverting recovered oxides into aluminum for secondary use. To contextualize present‐day limitations relative to future mitigation pathways, it is important to distinguish between the current carbon‐intensive configuration of the Hall–Héroult process and emerging low‐carbon alternatives. While conventional smelting remains dominated by electricity‐related emissions and carbon anode oxidation, inert‐anode‐based electrolysis offers the potential to eliminate direct GHG emissions. As noted, recent commercial‐scale demonstrations by ELYSIS have confirmed the technical feasibility of operating full‐size inert‐anode cells, indicating that direct CO_2_ and PFC emissions can be removed at industrial scale.

**FIGURE 7:**
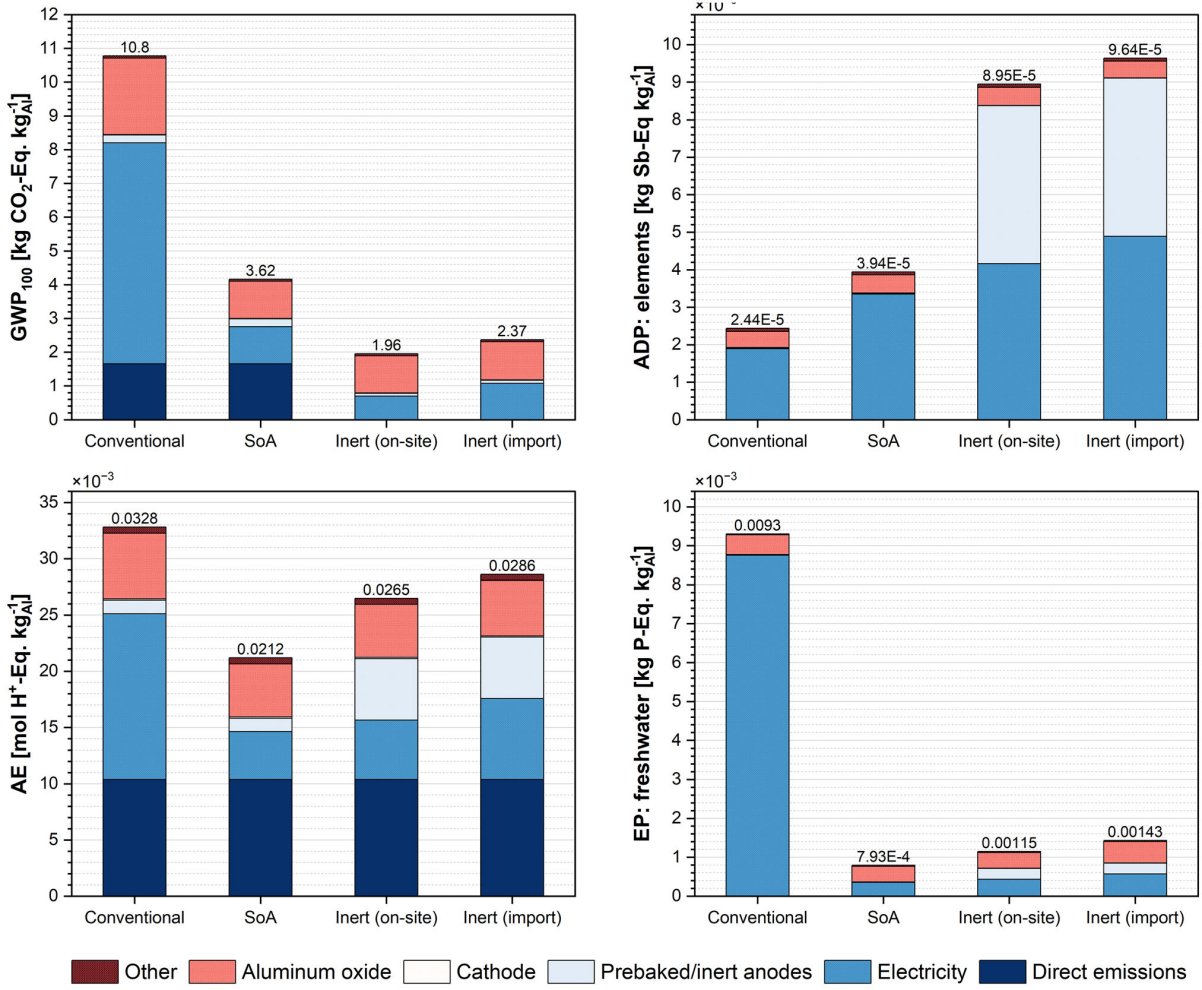
Impacts on selected impact categories over aluminum production scenarios. Note: Numbers above each column refer to total impacts per functional unit: 1 kg of primary aluminum, liquid at smelter gate.

A detailed investigation of the primary aluminum production impacts at the smelter gate is presented in Figure 7 for delineating the impacts associated with the producing 1 kg of primary aluminum. The impact assessment results for primary aluminum production under the considered scenarios emphasize the dominant contributions from electricity generation, Al_2_O_3_ supply via the Bayer process, and hard‐to‐mitigate direct emissions, particularly evident in the GWP_100_ and AE impact categories.

Regarding GWP, the integration of renewable electricity, implementation of enhanced energy efficiency measures in SoA smelters, and the adoption of cleaner heat sources in the Bayer process collectively yield an approximate 66% reduction in impacts between the conventional and SoA cases. Both the scenarios employ prebaked carbon anodes for electrolysis; while the anodes themselves are not the largest contributors, their consumption results in direct GHG emissions, thereby constraining the potential for achieving complete CO_2_ neutrality. As illustrated in both on‐site and import inert scenarios, the use of inert anodes eliminates direct GHG emissions. Despite a relative increase in electricity‐related impacts in these cases, a cumulative reduction of up to 82% in GWP_100_ impacts can be achieved. Similarly for EP, the transition to cleaner electricity sources results in a substantial reduction in impacts across all scenarios compared to the conventional baseline scenario. The impacts in the conventional baseline scenario stem mainly from fossil‐based electricity (94%) and bauxite residue management (5%). SoA scenario demonstrates the lowest overall EP impacts, achieving an 91% reduction relative to the baseline, whereas inert scenarios exhibit slightly higher values, primarily due to upstream copper and nickel mining and refining processes associated with inert anode production, as well as the increased energy consumption in these scenarios.

However, the ADP results reveal an opposite trend, with the baseline scenario exhibiting the lowest impacts and a progressive increase observed from SoA through inert (import) scenarios. This pattern can be attributed to the higher resource intensity of renewable energy technologies, which becomes more pronounced with the increased energy consumption in inert scenarios. Additionally, in these scenarios, the adoption of metal‐intensive inert anodes (particularly due to the use of copper and nickel) further amplifies the ADP impacts, resulting in impacts that are nearly four times higher than in the conventional scenario. In terms of acidification impacts, the AE results indicate a 35% reduction in SoA scenario compared to the conventional due to decreased cumulative SO_2_ and NO_*x*_ emissions resulting from the increased use of renewable energy sources. The direct process emissions remain the same across all scenarios. However, in inert scenarios, the introduction of inert anodes and the associated increase in energy consumption led to an increase in acidification impacts. Despite a marginal increase compared to the configuration, inert anode implementation may result in AE impact reductions of up to ≈20%. This case illustrates the so‐called ‘burden shifting’ phenomena. Nonetheless, a comprehensive evaluation of the AAB's environmental performance in LTES applications requires extending the analysis to include use‐phase impacts and comparing these with the benchmark technology, which is addressed in the next section.

### 3.2 Use Phase

**FIGURE 8:**
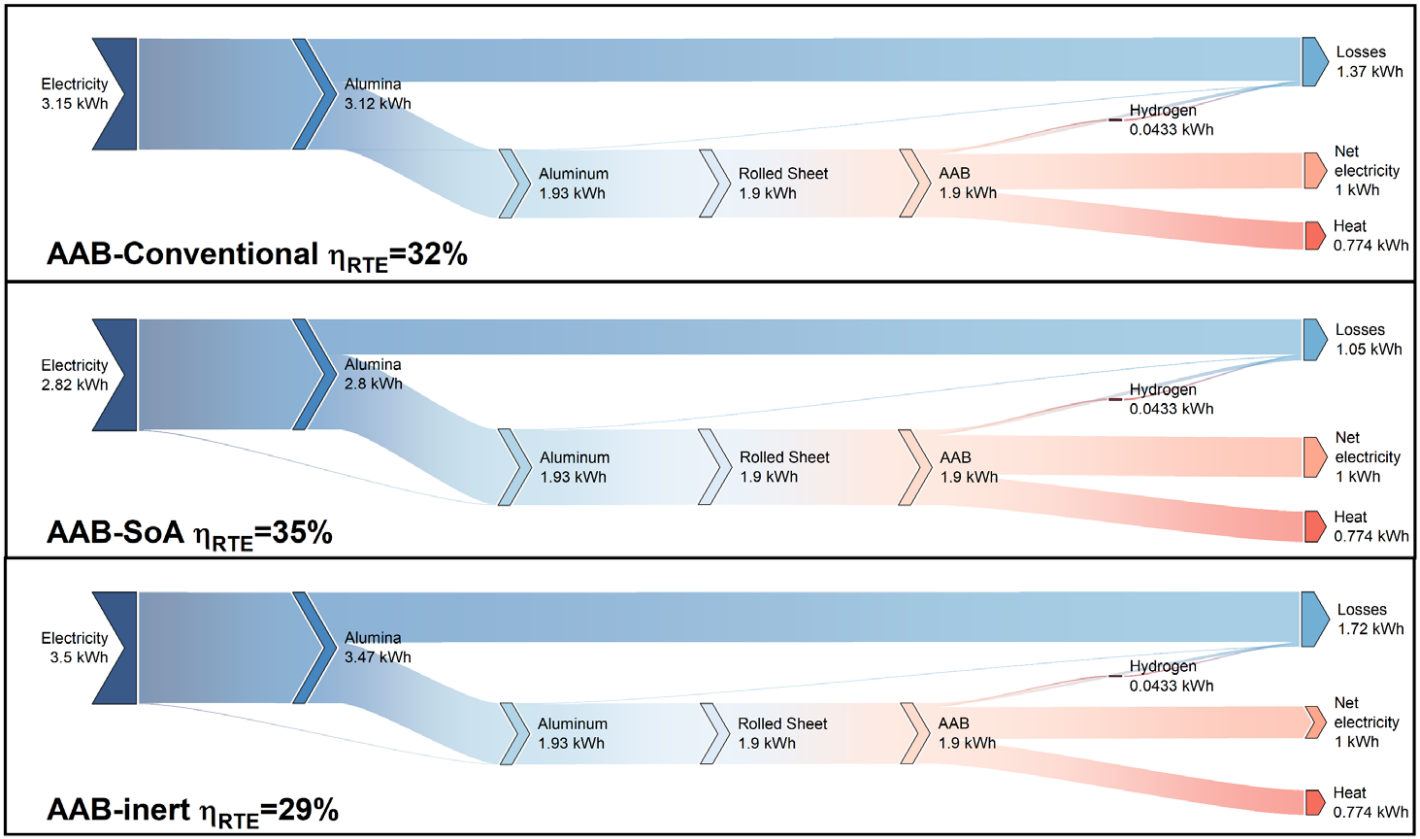
Sankey diagrams showing round‐trip conversion efficiencies over different scenarios for AAB LTES system.

**FIGURE 9:**
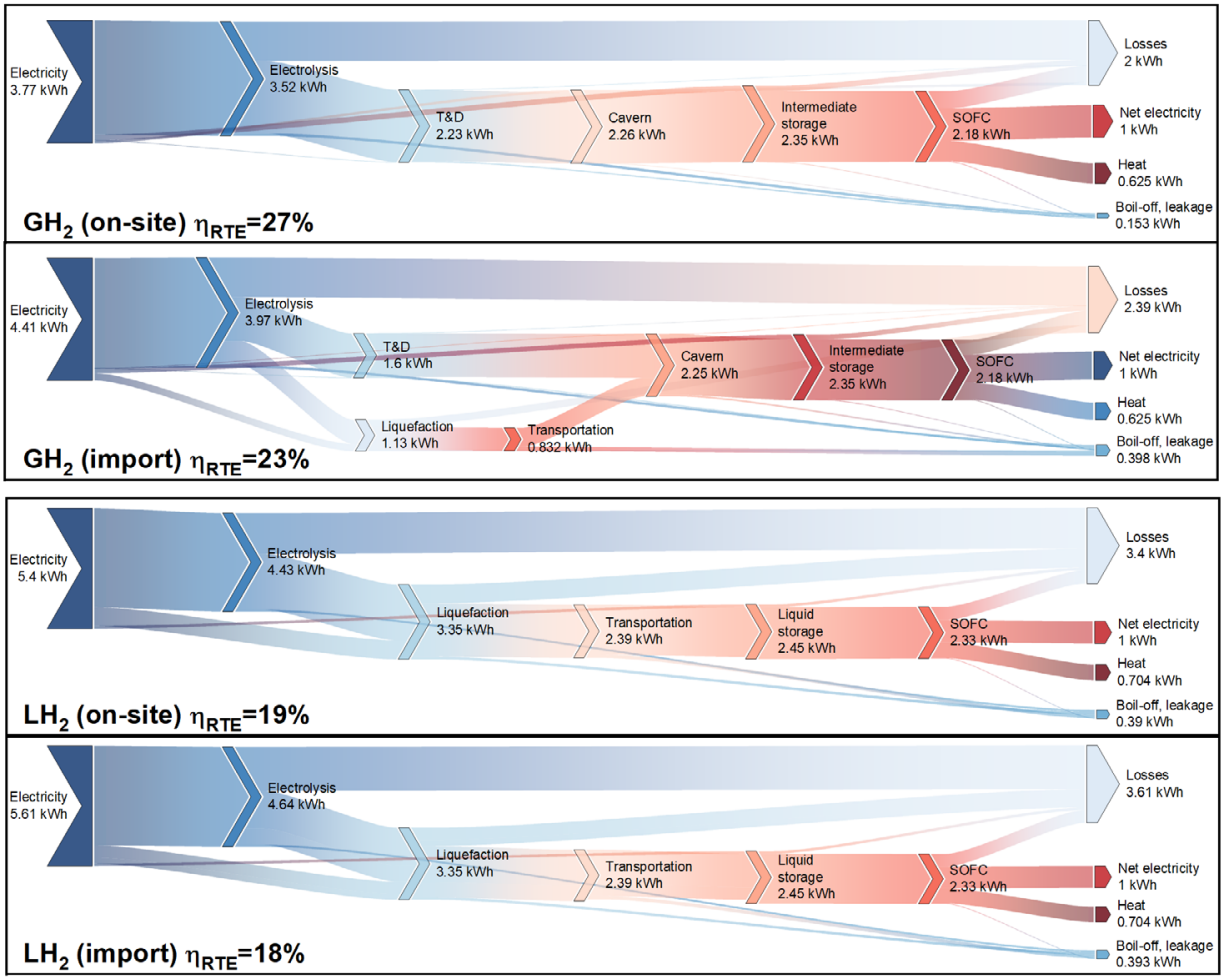
Sankey diagrams showing round‐trip conversion efficiencies over different scenarios for GH_2_ and LH_2_ based LTES systems.

Herein, the use‐phase performance of aluminum as a circular energy carrier is evaluated in comparison with H_2_. The analysis covers the entire energy conversion pathway, beginning with an energetic performance evaluation and followed by a comprehensive environmental impact assessment. To enable a comparative energetic analysis of both systems, Figures 8 and 9 present Sankey diagrams illustrating the energy flows across all scenarios. Each configuration delivers the same fixed electrical output of 1 kWh, while the thermal by‐products and total electricity inputs vary according to system efficiency and supply chain losses.

The conversion pathway for aluminum involves the use of electricity for the reduction of Al_2_O_3_ to aluminum, followed by the fabrication of rolled aluminum anode sheets, which are subsequently consumed in the AAB system. Unlike H_2_, aluminum anodes can be transported and stored without energy losses; therefore, transportation is not represented in the energy flow diagrams. The results reveal that SoA scenario achieves the highest RTE of 35%, primarily due to the reduced specific smelting energy demand representative of the SoA HH process. The baseline configuration attains a slightly lower RTE of 32%. In contrast, inert scenarios exhibit the lowest RTE at 29% both sharing identical conversion pathways. The decline in RTE is mainly attributed to the increased smelting energy intensity inherent to inert‐anode operation. Consequently, the adoption of inert anodes imposes an energy efficiency penalty during the aluminum regeneration stage.

For the H_2_ scenarios, electricity is used for electrolysis to produce gaseous hydrogen, which is then liquefied or compressed, transported, and stored prior to utilization in the SOFC‐GT system. These pathways account for boil‐off losses, leakage, and additional energy requirements arising from the respective supply chains (see Figure 9). When compared with LH_2_, AAB‐based LTES demonstrates a distinct efficiency advantage. Across all configurations, the AAB system outperforms LH_2_ by at least 2% in RTE. In LH_2_ pathways, AEC requires around 51.8 kWh_e_ per kg H_2_. Downstream processes such as compression, liquefaction, and re‐liquefaction add further energy requirements, increasing the cumulative losses. As evidenced by the energy‐flow comparisons, on‐site underground storage achieves the highest RTE at 27%, whereas increasing the share of LH_2_ reduces RTEs to as low as 18%, highlighting hydrogen's sensitivity to the handling pathway under baseline assumptions.

**FIGURE 10:**
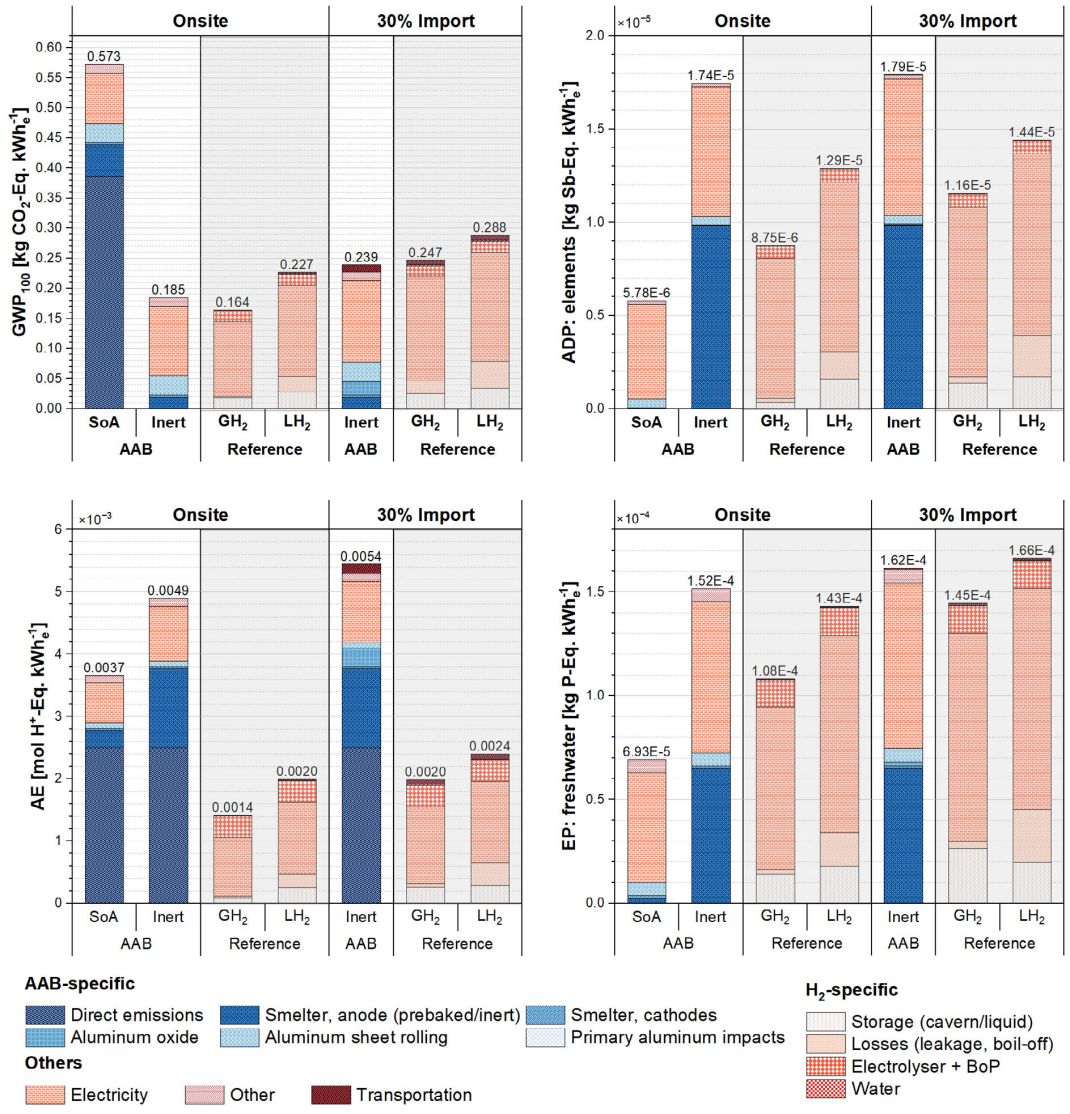
Impact comparison of selected impact categories for net 1 kWh_e_ stored using fabricated aluminum anodes and H_2_. (Note: Numbers above of each column refer to total impacts per functional unit.).

Based on the energetic analysis of the different scenarios for both systems, the use‐phase impacts associated with the fabricated aluminum anodes and H_2_ are evaluated and presented in Figure 10 for the selected impact categories. For the AAB system, introduction of inert anodes in AAB‐inert on‐site and import scenarios, direct GHG emissions are eliminated, leading to up to 68% reduction in GWP_100_ impacts resulting in 0.185–0.239 kg CO_2_‐eq kWh_e_^−1^. Owing to its superior RTE, AAB‐inert delivers 7.5–30% lower energy‐related impacts in the on‐site scenario. As the share of imported LH_2_ increases, the difference widens to 57%, making on‐site AAB inherently competitive even relative to GH_2_ stored in underground storage. Impacts from aluminum ingot casting, sheet rolling, and the transport of both aluminum and oxides emerge as hotspots; addressing these stages could further reduce GWP.

In ADP, AE, and EP, the metal‐intensive composition of inert anodes emerges as the key driver of increased impacts, with ADP roughly tripling compared to the prebaked anode configuration. This outcome underscores a clear resource trade‐off associated with decarbonization. While inert anodes eliminate direct emissions, they substantially increase the demand for critical raw materials and the associated ADP and EP burdens. Transportation contributes a secondary but non‐negligible share in AAB‐inert import scenario, where shipping aluminum anodes from Saudi Arabia to Germany and returning aluminum oxides accounts for 14% of total GWP impacts. This transboundary supply chain amplifies the system's overall footprint despite the use of renewable electricity in production.

For the reference H_2_ systems, in total electricity consumption, handling and storage‐related effects dominate all impact categories being less dominant when hydrogen is handled and stored in gas form due to elimination of additional boil‐off and leakages, liquefaction and re‐liquefaction energy consumption. In GWP_100_ and AE, these electricity‐related emissions, along with conversion and storage H_2_ losses, account for the major share of the impacts where LH_2_ based system demonstrates higher impacts. Regarding infrastructure‐related impacts, the electrolyzer and BoP exhibit a relatively high contribution to the AE and EP impact categories. In ADP and EP, the mineral and resource burdens of renewable electricity generation that are also indirectly represented in hydrogen losses contribute significantly to total impacts. The observed differences between the on‐site and import H_2_ are due to supply chain and standby losses. Notably, particularly in the LH_2_ case, accommodating hydrogen in the storage tank for extended durations during DF period causes higher impacts. Compared with AAB, transportation impacts remain smaller in LH_2_ scenarios due to hydrogen's higher gravimetric energy density, which allows for more energy transported per unit mass. All in all, GH_2_ underground storage outperforms both AAB‐ and LH_2_‐based systems in the onsite case. Nevertheless, AAB is highly competitive against GH_2_ in import scenario where it outperforms both GH_2_ and LH_2_ in GWP impacts.

### 3.3 Cradle‐to‐Use

**FIGURE 11:**
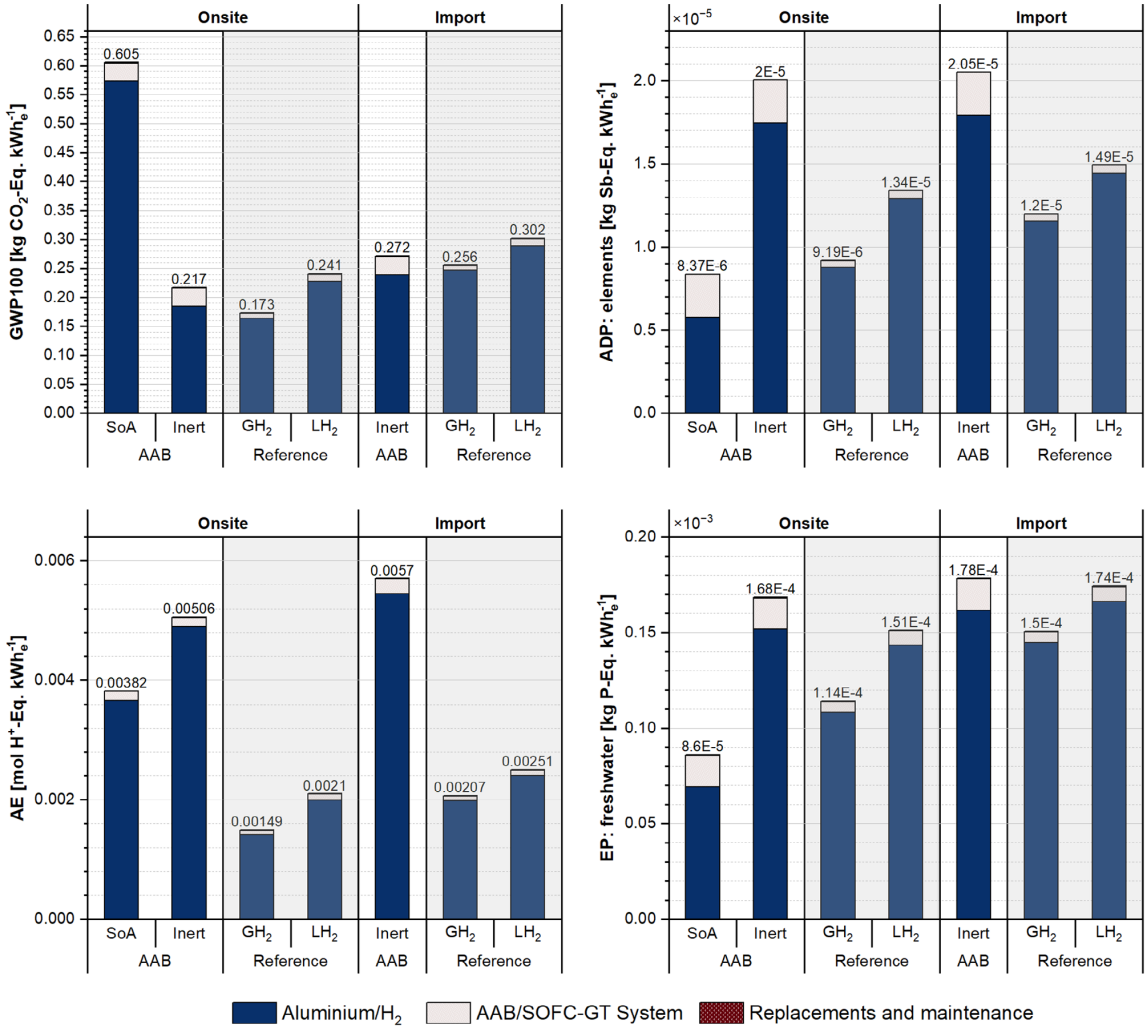
Comparison of cradle‐to‐use impacts on selected impact categories for net 1 kWh_e_ stored. (Note: Numbers above each column refer to total impacts per functional unit.).

In this section, the cradle‐to‐use impacts are discussed comparatively, providing a holistic overview by incorporation of the cradle‐to‐gate performance of the conversion technologies and a detailed examination of the aluminum anodes and H_2_ supply chains during the use phase, as illustrated in Figure 11. These aspects, discussed in detail in the previous sections, are now brought together to present the total system impacts. The breakdown of the results verifies that the major share of the total impacts is associated with the aluminum anodes in the AAB system, and the H_2_ supply chain in the reference system.

GH_2_ underground storage provides the best overall performance across impact categories when aluminum is produced with inert anodes. The AAB‐SoA scenario records the lowest ADP and EP, yet direct emissions prevent this advantage in GWP and AE. In GWP, inert anodes improve AAB's environmental performance, but the opposite occurs in the other impact categories. When cradle‐to‐gate impacts are included, AAB becomes highly comparable with LH_2_, emphasizing the need to scrutinize AAB‐system related impacts. Furthermore, for both technologies, the replacement and maintenance contribute only a minor fraction to the total impacts. The higher environmental performance of H_2_ alternatives is particularly evident in ADP and AE in both on‐site and import configurations. As the storage duration of LH_2_ has a strong influence on GWP outcomes, potential improvements in the AAB technology might result in even better performance than LH_2_ and very competitive against GH_2_ depending on the supply chain. This underscores the importance of use‐case sensitivity in assessing H_2_‐based storage particularly for GWP impacts.

### 3.4 Uncertainty Analysis

**FIGURE 12:**
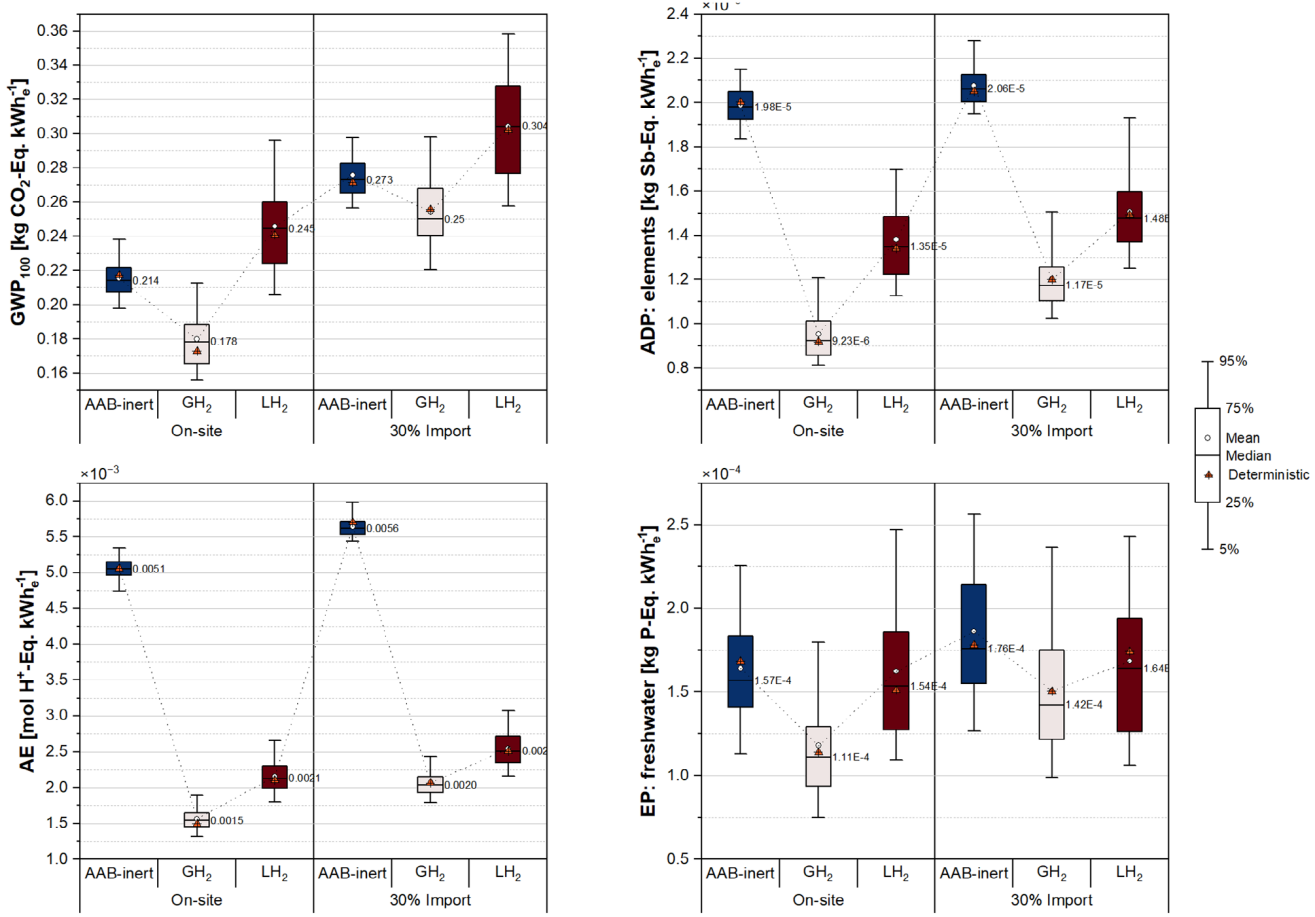
Uncertainty analysis of the selected impact categories.

An uncertainty analysis was performed using a Monte Carlo simulation (*n* = 100) to propagate parametric uncertainty across cradle‐to‐use system boundaries for both AAB‐ and H_2_‐based LTES systems. The probability distributions were implemented to capture variability in key model parameters and their influence on the overall environmental performance. Since the use phase impacts dominate the total impacts across all selected impact categories, as shown in the cradle‐to‐use results, this section focuses primarily on the uncertainties associated with the use phase parameters. Nonetheless, the Monte Carlo results presented in Figure 12 incorporate all uncertainties defined for both the cradle‐to‐gate and use phase stages. In all figures, the circular markers denote the mean values, while the box‐whisker ranges represent the distribution of outcomes based on propagated uncertainties. The red triangular markers denote the deterministic estimates presented in the cradle‐to‐use results section, included here for reference within the box‐and‐whisker plot.

The probabilistic results reflect the influence of key uncertain parameters in each LTES system. For the AAB system, uncertainty stems primarily from inert‐anode lifetime, electrolysis electricity intensity, and total system lifetime. LH_2_ uncertainty is dominated by electrolyzer electricity variation, utilization efficiency, storage duration, and cumulative boil‐off and handling losses. GH_2_ uncertainty is comparatively lower, as its main variability originates from storage performance and electricity consumption. Across all systems and categories, deterministic values fall near the distribution mean and median, confirming that the single‐point estimates represent plausible central tendencies.

In the on‐site case, GH_2_ with underground storage demonstrates the lowest median GWP (0.176 kg CO_2_‐eq   kWh^−1^), with a 5th–95th percentile spread of ≈35%, indicating moderate but manageable variability. AAB‐inert exhibits a higher median (≈0.214 CO_2_‐eq kWh^−1^), yet its 5th percentile lies within the GH_2_ distribution, meaning that while AAB is unlikely to outperform GH_2_, low‐end outcomes may approach GH_2_ performance. Relative to LH_2_, AAB performs more consistently. LH_2_ has the highest uncertainty, driven majorly by boil‐off and extended storage requirements. Its median GWP is nearly 10% higher than the deterministic value (0.225 kg CO_2_‐eq kWh^−1^), and the wide distribution indicates considerable sensitivity to operational conditions. The AAB median falls within the 5th–25th percentile range of GH_2_, confirming AAB's systematically lower climate burden under on‐site production. When 30% of the hydrogen must be imported in liquid form, uncertainties increase most notably for GH_2_ because imported LH_2_ introduces additional loss pathways and transport energy penalties. Nevertheless, AAB exhibits a more compact uncertainty range, suggesting lower probability of exceeding GH_2_ impacts. When contrasted with LH_2_, the AAB median remains lower, but the distributions overlap, indicating that under unfavorable AAB or favorable LH_2_ conditions, comparative ranking may shift. This overlap underscores the role of supply chain configuration in determining climate competitiveness.

Across both scenarios, the ADP distributions are clearly separated among the alternatives. AAB‐inert consistently shows substantially higher ADP due to inert anode consumption. GH_2_ and LH_2_ demonstrate considerably lower values with narrower spreads. The lack of distribution overlap indicates a robust ranking: AAB invariably performs worse in terms of mineral resource depletion, independent of scenario or uncertainty propagation under given considerations. A similar pattern emerges in the AE category. AAB‐inert is associated with the highest impacts and exhibits no overlap with GH_2_ or LH_2_ distributions in either the on‐site or import conditions. The magnitude of the difference is even more pronounced than in ADP, reflecting the strong influence of aluminum smelting emissions. GH_2_ and LH_2_ remain closely aligned, although LH_2_ generally displays a larger uncertainty range linked to storage and loss variability. EP is characterized by broader uncertainty bands across all alternatives relative to the other categories. Under on‐site conditions, GH_2_ again performs best, with a lower median and narrower interquartile range. However, AAB and LH_2_ show overlapping percentile ranges, indicating that their relative positions are more uncertain. In the import scenario, uncertainties expand further and partial overlaps emerge for all three systems, signaling that EP outcomes are more sensitive to upstream supply chain assumptions than the other impact categories. No alternative is clearly dominant under import conditions.

## 4 Discussion

The results are discussed here to synthesize the key findings and interpret the implications of the comparative exploratory assessment of AAB and H_2_‐based LTES systems. The discussion focuses on identifying environmental hotspots, evaluating the influence of decarbonization scenarios, and exploring the sustainability potential of AAB technology relative to H_2_ as a reference system.

The manufacturing impacts of AAB currently exhibit lower environmental performance compared with the SOFC‐GT system. Although these manufacturing impacts are not the dominant contributors under cradle‐to‐use conditions, targeted improvements could substantially strengthen the competitiveness of AAB. For instance, a 50% reduction in manufacturing‐related impacts would already allow AAB to surpass hydrogen import scenarios in terms of GWP and bring its performance closer to GH_2_ in the on‐site case. Such improvements would also enhance AAB's performance relative to GH_2_ in the EP category and could render it competitive with GH_2_, albeit with some trade‐offs. Within the system‐related impacts, sodium stannate used in the electrolyte emerges as a hot spot; its substitution could achieve modest reductions in ADP impacts. By contrast, impacts in AE are comparatively minor and would not significantly alter the overall comparison. More broadly, the hotspots identified in the cradle‐to‐gate assessment namely the electrolyte, electrolyte tank, cables, and cell housing warrant focused design modifications and material substitution to achieve a more environmentally favorable AAB configuration.

Under cradle‐to‐use conditions, aluminum in the AAB system and hydrogen production and storage in H_2_‐based LTES systems are the dominant contributors to environmental impacts. For AAB, substantial improvements could be achieved through reduced electricity consumption in HH process employing inert anodes, extended inert‐anode lifetimes, and lower copper content in anode formulations; together, these modifications would yield noticeable reductions across all impact categories. When GWP is used as the primary decision criterion, AAB offers several advantages over hydrogen, particularly its ease of handling and higher efficiency. Nevertheless, the fabrication of aluminum into anode sheets remains a considerable contributor to GWP, prompting reconsideration of the aluminum feedstock used.

For hydrogen‐based alternatives, GH_2_ remains promising due to its high gravimetric energy density, provided that underground storage and an efficient supply chain are ensured. By contrast, the results indicate that LH_2_ offers benefits for transport relative to GH_2;_ however, its environmental performance in LTES applications is bound with boil‐off and standby losses. Overall, owing to its long‐term storability and high energy density, AAB presents a competitive alternative to hydrogen‐based systems, particularly with respect to GWP. The efficiency advantage of AAB is outweighed by the material‐ and process‐related burdens linked to inert anode manufacture and HH process in other categories highlighting the improvement areas.

## 5 Conclusions

This study presented an exploratory LCA of primary a primary AAB based on an early‐stage design configuration, conducted as a forward‐looking analysis to evaluate its environmental performance and associated trade‐offs, benchmarked against two H_2_‐based systems within LTES context. From a technical performance perspective, the RTE of the AAB system provides strong motivation for its further development, particularly when combined with its high volumetric energy density, comparatively simple handling for storage and transport, and potential for circular material utilization. These motivating aspects, in combination with prospective low‐carbon aluminum production scenarios, translate into competitive environmental performance, particularly with respect to climate change impacts. Nevertheless, the analysis identifies several technological development hotspots that must be addressed to ensure that this conditionally sustainable configuration becomes genuinely sustainable in practice. This requires not only targeted technological improvements but also the careful and consistent implementation of measures within future aluminum production pathways.

When assessing the performance of the AAB within the cradle‐to‐gate boundaries, no single component dominates across the four impact categories considered. However, consistent hotspots are identified in the electrolyte tank, electrolyte, copper cables, and cell housing. As discussed in the previous section, the elevated impacts associated with these components originate mainly from upstream PVC, KOH, and polyester resin production, as well as copper extraction and refining. These findings indicate clear leverage points for targeted design optimization and material substitution. Increasing recycled content and dose minimization of chemical inputs represent direct abatement opportunities across all categories. A substantial improvement in the environmental performance of system components could render AAB highly competitive. This confirms that the forward‐looking objective of this study, namely, the identification of key development pathways, has been successfully achieved.

A clear observation is the dominance of use‐phase impacts, underscoring the decisive influence of RTE, electricity source and system lifetime on overall performance. The AAB system achieves a RTE of 29–35%, which is at least 2% higher than GH_2_ and about 10% higher than LH_2_. This higher efficiency reduces energy losses and consequently lowers their contribution to total life cycle impacts. Furthermore, assuming decarbonized power supply and inert anode technology for aluminum production, a GWP reduction of ∼66% is proven feasible relative to the conventional case. Nevertheless, the environmental benefits of higher RTE and decarbonization are offset by increased impacts in ADP and EP, primarily driven by the material intensity of metallic inert anodes. Additional burdens arise from, the direct process emissions, and transportation requirements, particularly in import scenario, where cross‐border logistics of aluminum and oxides contribute up to 14% of total GWP. Moreover, the use of PV‐based electricity and higher metal demand further increases in ADP, EP, and AE, reflecting clear burden shifting effects. These findings emphasize the need for an integrated lifecycle optimization approach that combines renewable energy deployment with low‐impact anode chemistries, improved material efficiency and circular material flows.

Ultimately, GH_2_ outperforms AAB across all categories when inert scenarios are considered, although the AAB remains highly competitive in terms of GWP. In the import case, the aluminum anode itself shows lower GWP than GH_2_. However, once the full AAB system related impacts are included, total GWP exceeds that of GH_2_. In contrast, the AAB performs better than LH_2_ in terms of GWP. These results underline the need for further optimization of the AAB system. The SoA scenario performs best in ADP and EP, yet exhibits the highest impacts in GWP due to direct emissions. This demonstrates that no single pathway is environmentally superior across all categories. Instead, achieving the full sustainability of AABs requires a balance approach combining lower energy intensity, renewable integration, and the development of resource‐efficient and durable inert anodes.

Moreover, the uncertainty analysis confirms the deterministic ranking but shows that LH_2_ actually has the widest GWP and ADP uncertainty range, while AAB's GWP and ADP bands are narrower and controlled mainly by aluminum production energy and anode lifetime. Despite a higher median GWP than GH_2_, AAB's lower‐end outcomes occasionally approach GH_2_ performance, while it consistently outperforms LH_2_. GH_2_ remains the most stable option overall. Across all scenarios, AAB exhibits substantially higher ADP and AE impacts, with nonoverlapping distributions, whereas EP outcomes show broader uncertainty and partial overlaps, particularly under import conditions. Overall, the comparative results underline a dual challenge for AAB development: achieving substantial climate benefits without exacerbating resource depletion or ecosystem impacts. Addressing this requires a holistic strategy that couples renewable electricity with material circularity, optimized logistics, and validated component durability to ensure that upstream burdens do not offset use‐phase benefits.

To enhance competitiveness and sustainability, future work should focus on:

• Reducing or replace hotspot materials by adopting recycled or bio‐based polymers, lower‐impact tank resins, optimized electrolytes, and use of secondary materials.
• Advancing aluminum smelting technologies, emphasizing renewable integration, process efficiency, and low‐impact inert anode manufacturing.
• Closing material loops and rationalizing logistics by minimizing transboundary aluminum/Al_2_O_3_ flows, quantifying losses, and integrating recovery and remanufacturing routes.
• Expanding the modeling scope to incorporate the feeding assembly, material and electrolyte losses, and the allocation of co‐generated heat and hydrogen, informed by empirical data from large‐scale operation.
• Examining regional and temporal sensitivities, especially for LH_2_ storage duration and AAB transport networks, under prospective low‐carbon energy scenarios.

Collectively, these measures will enable a more robust and sustainable deployment of aluminum‐based storage systems, supporting their potential to complement or partially substitute hydrogen‐based LTES solutions in a decarbonized, resource‐efficient energy future.

## Supporting Information

Additional supporting information can be found online in the Supporting Information section.

## Author Contributions

**Hüseyin Ersoy**: conceptualization, methodology, investigation, formal analysis, data curation, visualization, writing – original draft. **Friedrich B. Jasper**: methodology, investigation, validation, writing – review & editing. **Christina Wulf**: methodology, validation, hydrogen system modeling, writing – review & editing. **Stefano Passerini**: supervision, writing – review & editing. **Manuel J. Baumann**: supervision, methodology, writing – review & editing. **Marcel Weil**: supervision, writing – review & editing. **Tomás B. Ramos**: supervision, methodology, writing – review & editing.

## Funding

This study was supported by Karlsruhe Institute of Technology (KIT Future Fields Stage II) and Deutsche Forschungsgemeinschaft (390874152).

## Supporting information

Supplementary Material

## Data Availability

The data that support the findings of this study are available in the supplementary material of this article.
