# Supplementary material for: Exploring the Environmental Sustainability of Primary Al–Air Batteries for Long‐Term Energy Storage Applications
Source: ChemSusChem. 2026 Apr 19;19(8):e202502714. doi: 10.1002/cssc.202502714 (PMC13092360; doi:10.1002/cssc.202502714)
Supplement: Supplementary file 1 — Supplementary Material [file CSSC-19-e202502714-s001.pdf]

The Effects of γ Phase on Superelasticity in Cu-Al-Mn Alloys Fabricated via Laser Powder Bed Fusion

Additional supporting information can be found online in the Supporting Information section. **Supporting Table S. 1.** System parameter ranges and distributions. **Supporting Table S. 2.** Common parameter ranges and distributions. **Supporting Table S. 3.** Life cycle inventory of primary AAB system. Reference flow is written in bold. **Supporting Table S. 4.** LCI of air cathode fabrication. (Spraying efficiency (eff_spray) =60%). **Supporting Table S. 5.** Life cycle inventory of α-MnO_2. Reference flow is written in bold[1]. **Supporting Table S. 6.** Life cycle inventory of Freudenberg H23C2. Reference flow is written in bold. **Supporting Table S. 7.** Life cycle inventory of electrolyte. Reference flow is written in bold. **Supporting Table S. 8.** Life cycle inventory of sodium stannate. Reference flow is written in bold. **Supporting Table S. 9.** Life cycle inventory of electrolyte tank. Reference flow is written in bold. **Supporting Table S. 10.** Life cycle inventory of cable. Reference flow is written in bold. **Supporting Table S. 11.** Life cycle inventory of cell housing. Reference flow is written in bold. **Supporting Table S. 12.** Life cycle inventory of steel pipe. Reference flow is written in bold. **Supporting Table S. 13.** Life cycle inventory of FKM gasket. Reference flow is written in bold. **Supporting Table S. 14.** Life cycle inventory of stack frame. Reference flow is written in bold. **Supporting Table S. 15.** Life cycle inventory of steel frame, material. Reference flow is written in bold. **Supporting Table S. 16.** Life cycle inventory of steel frame, processing. Reference flow is written in bold. **Supporting Table S. 17.** Life cycle inventory of T-profile welding. Reference flow is written in bold. **Supporting Table S. 18.** Life cycle inventory of heat exchanger. Reference flow is written in bold. **Supporting Table S. 19.** Life cycle inventory of pump. Reference flow is written in bold. **Supporting Table S. 20.**

Life cycle inventory of PCS. Reference flow is written in bold. **Supporting Table S. 21.** Life cycle inventory of BMS. Reference flow is written in bold. **Supporting Table S. 22.** Life cycle inventory of stack monitoring device. Reference flow is written in bold. **Supporting Table S. 23.** Life cycle inventory of secondary aluminum at smelter (liquid). Reference flow is written in bold. (recycled_Al2O3=share of recycled content). **Supporting Table S. 24.** Life cycle inventory of aluminium anode rolled sheet. Reference flow is written in bold. **Supporting Table S. 25.** Life cycle inventory of sheet rolling process. Reference flow is written in bold. **Supporting Table S. 26.** Life cycle inventory of inert anodes. Reference flow is written in bold. **Supporting Table S. 27.** Life cycle inventory of AAB use phase. Reference flow is written in bold. **Supporting Table S. 28.** Life cycle inventory of alkaline water electrolysis in Germany. Reference flow is written in bold. **Supporting Table S. 29.** Life cycle inventory of alkaline water electrolysis in Saudi Arabia. Reference flow is written in bold. **Supporting Table S. 30.** Life cycle inventory for gaseous hydrogen, transmission and distribution, prior underground storage. Reference flow is written in bold. **Supporting Table S. 31.** Life cycle inventory for hydrogen liquefaction, prior transport. Reference flow is written in bold. **Supporting Table S. 32.** Life cycle inventory for transportation of liquid hydrogen within Germany. Reference flow is written in bold. **Supporting Table S. 33.** Life cycle inventory for transportation of liquid hydrogen from Saudi Arabia. Reference flow is written in bold. **Supporting Table S. 34.** Life cycle inventory for underground storage operation. Reference flow is written in bold. Supporting **Table S. 35.** Life cycle inventory intermediate storage tank manufacture. Reference flow is written in bold. **Supporting Table S. 36.** Life cycle inventory for intermediate storage tank. Reference flow is written in bold. **Supporting Table S. 37.** Life cycle inventory of SOFC-GT operation (underground storage). Reference flow is written in bold. **Supporting Table S. 38.** Life cycle inventory of SOFC-GT operation (liquid storage). Reference flow is written in bold. **Supporting Table S. 39.** Summary of electricity mix. **Supporting Table S. 40.** AAB battery system cradle-to-use GWP100 impact assessment results.

Functional unit: 1 kWh of storage capacity. **Supporting Table S. 41.** AAB battery system cradle-to-use ADP: elements impact assessment results. Functional unit: 1 kWh of storage capacity. **Supporting Table S. 42.** AAB battery system cradle-to-use AE impact assessment results. Functional unit: 1 kWh of storage capacity. **Supporting Table S. 43.** AAB battery system cradle-to-use EP: freshwater impact assessment results. Functional unit: 1 kWh of storage capacity. **Supporting Table S. 44.** AAB battery system cradle-to-use AE impact assessment results. Functional unit: 1 kWh of storage capacity. **Supporting Table S. 45.** AAB cradle-to-use impact assessment results across considered scenarios. (IAI: EF V3.1). **Supporting Table S. 46.** Hydrogen cradle-to-use impact assessment results across considered scenarios. (IAI: EF V3.1). **Supporting Table S. 47.** AAB-inert (on-site) scenario cradle-to-use impact uncertainty analysis percentiles. (Number of iterations: 100). **Supporting Table S. 48.** AAB-inert (import) scenario cradle-to-use impact uncertainty analysis percentiles. (Number of iterations: 100). **Supporting Table S. 49.** GH_2 (on-site) scenario cradle-to-use impact uncertainty analysis percentiles. (Number of iterations: 100). **Supporting Table S. 50.** GH_2 (import) scenario cradle-to-use impact uncertainty analysis percentiles. (Number of iterations: 100). **Supporting Table S. 51.** LH_2 (on-site) scenario cradle-to-use impact uncertainty analysis percentiles. (Number of iterations: 100). **Supporting Table S. 52.** LH_2 (import) scenario cradle-to-use impact uncertainty analysis percentiles. (Number of iterations: 100).
